# Microtubule disruption changes endothelial cell mechanics and adhesion

**DOI:** 10.1038/s41598-019-51024-z

**Published:** 2019-10-17

**Authors:** Andreas Weber, Jagoba Iturri, Rafael Benitez, Spela Zemljic-Jokhadar, José L. Toca-Herrera

**Affiliations:** 1 Institute for Biophysics, Department of Nanobiotechnology, University of Natural Resources and Life Sciences Vienna, Muthgasse 11, A-1190 Vienna, Austria; 2 Dpto. Matemáticas para la Economía y la Empresa, Facultad de Economía, Universidad de Valencia, Avda. Tarongers s/n, 46022 Valencia, Spain; 3 Department of Biophysics, Medicine Faculty, University of Ljubljana, Vrazov trg 2, 1000 Ljubljana, Slovenia

**Keywords:** Applied physics, Biological physics, Cytoskeleton, Biomaterials - cells, Biophysics, Cell biology, Materials science, Physics

## Abstract

The interest in studying the mechanical and adhesive properties of cells has increased in recent years. The cytoskeleton is known to play a key role in cell mechanics. However, the role of the microtubules in shaping cell mechanics is not yet well understood. We have employed Atomic Force Microscopy (AFM) together with confocal fluorescence microscopy to determine the role of microtubules in cytomechanics of Human Umbilical Vein Endothelial Cells (HUVECs). Additionally, the time variation of the adhesion between tip and cell surface was studied. The disruption of microtubules by exposing the cells to two colchicine concentrations was monitored as a function of time. Already, after 30 min of incubation the cells stiffened, their relaxation times increased (lower fluidity) and the adhesion between tip and cell decreased. This was accompanied by cytoskeletal rearrangements, a reduction in cell area and changes in cell shape. Over the whole experimental time, different behavior for the two used concentrations was found while for the control the values remained stable. This study underlines the role of microtubules in shaping endothelial cell mechanics.

## Introduction

Eukaryotic cells are complex biological systems featuring high hierarchical order with respect to their structure, function and form. Cells are known to interact with their surroundings not only via chemical or biochemical signals, but also through their ability to sense, transduce and exert (mechanical) forces^1^. In recent years, studying cell mechanical properties has gained an increasing interest. For instance, studies have shown that cellular response, biology and fate highly depend on mechanical features of the underlying substrate^2^. Variations in cell mechanical properties are indicators of changes in the cellular metabolism or state (e.g. disease, cancer, age, …), and can, be used as diagnosis tool^3,4^. In addition, knowledge of complex cellular transformations, such as the epithelial to mesenchymal transitions, can be deepened by following changes in cell mechanics^5^.

First studies regarding cell mechanical properties tackled an important question still under discussion: the role that different cellular features like membranes, cytoskeletal components and nucleus play in defining the mechanical response^6^. The unraveling of which cytoskeletal component had the most prominent role in cell mechanics was also of main interest. Rotsch *et al*. were one of the first groups to study this behavior extensively, stating that cell mechanics (in their case Young’s Modulus) mostly depends on the actin filaments while microtubules play only a minor role^7^. More recently, different works have underlined the role of microtubules in cell mechanics^8,9^. Microtubules play a prominent role in mitosis, intracellular transport, the formation of cilia and flagella, developmental biology, focal adhesion formation, and many other processes^10^. They have particularly interesting polymerization and depolymerization kinetics that can be targeted externally by chemical agents^11^. Targeting the microtubules with e.g. colchicine leads to rapid depolymerization, followed by changes in the expression of genes associated to migration, growth, adhesion and inflammation^12^ – thus also further changes in cell mechanical properties are expected. Other agents interacting with microtubules include nocodazole and colcemide (both hindering filament polymerization), taxol (which stabilizes microtubules) or recent synthetic drugs such as cryptophycins. The different drugs are often used in cell biological studies to stall cells in the mitotic phase but also in cancer therapy; their effect on cellular mechanics has been the focus of various studies.

In addition, one has to consider that a cell is a living organism where its different constituents interact dynamically with each other. With respect to cell mechanics, actin filaments have received most of the attention in recent years, because of their roles in cell movement, cell shape and cell architecture. Nevertheless, the crosstalk between microtubules and the actin network has been extensively studied^1,13–15^. The interaction of these two cytoskeletal components is led by different mechanisms, e.g. crosslinking, guidance of filament growth, anchoring of microtubules by actin networks or actin nucleation from microtubule plus ends. Therefore, the changes in the microtubule network by e.g. disruption can also lead to variations in the properties of the actin network. Most prominently, several groups have reported that depolymerization of microtubules induces actin polymerization, promoting the formation of actin stress fibers^16–20^.

Atomic force microscopy (AFM) is today an established tool for measuring cell mechanics^21,22^. Other suitable techniques include optical and magnetic tweezers, surface force apparatus, and micropipette aspiration techniques^23^. AFM works by probing a sample with a tip (the tip end radius can be as small as a few nm) that is positioned at the end of a cantilever. Due to interactions between the tip and the sample surface (e.g. from van der Waals or electrostatic forces, but also by repulsion in the contact regime), the cantilever bends. This bending can be quantified and therefore the strength of the interaction between tip and sample is measured. This allows for topographical measurements with nearly nanometric resolution and can be additionally used in force spectroscopy mode to probe material mechanics when in contact^24^. The versatility of this technique concerning tip geometry, chemistry, and measurement modes while simultaneously allowing to measure in the native state (e.g. liquid environment, T = 37 °C) is one of the major advantages. Furthermore, it can be combined with different optical microscopy and spectroscopy set-ups^25^. Generally, most research works on cell mechanics using AFM are concerned with measuring the apparent Young’s Modulus of biomaterials by indentation and then using the Hertz model (with Sneddon extension for non-spherical indenters) for data quantification^26,27^. Some interesting work, e.g. on the differentiation of aggressiveness of cancer cell lines has been done by this approach^28^. In recent years, additional models describing the viscoelastic cellular response to mechanical stresses have been developed^29^. One approach to model this behavior is to reduce the cellular complexity to a set of various elastic and plastic components (springs and dashpots), set in parallel. In this way, the cell is modeled as the sum of the subcellular components^29^. Even more recently, such approaches have been combined in a force-mapping set-up, thus allowing mapping of the viscoelastic properties of cells^30–32^.

This study aims to further examine the influence of microtubules on cytomechanics and tip-cell-adhesion properties of HUVEC cells, using AFM in force spectroscopy mode. The influence of the microtubules was determined by depolymerization, using colchicine at different concentrations (0.1 mM and 2 mM). Young’s Modulus and cell relaxation were determined as indicators for cell deformability and rheological properties. Furthermore, the adhesion between the AFM tip and the cell (force, rupture events) and the cellular area were determined. Changes in the cytoskeleton were followed by fluorescence microscopy after staining the microtubules, the actin filaments and the nucleus.

For both concentrations, after already 30 min a stiffening of the cells accompanied by longer relaxation times (indicating a reduction in fluidity) was found. In addition, the adhesion between tip and cell was reduced, hinting towards changed membrane properties (or membrane-cytoskeleton connection). Fluorescence microscopy showed a rearrangement of the cytoskeleton, and a reduction in cell area and changes in cellular shape. The cell mechanical properties for the low concentration over time were similar to the control values, while for the higher concentration significant differences were observed.

## Results

### Microtubule depolymerization and cell morphological changes

**Figure 1:**
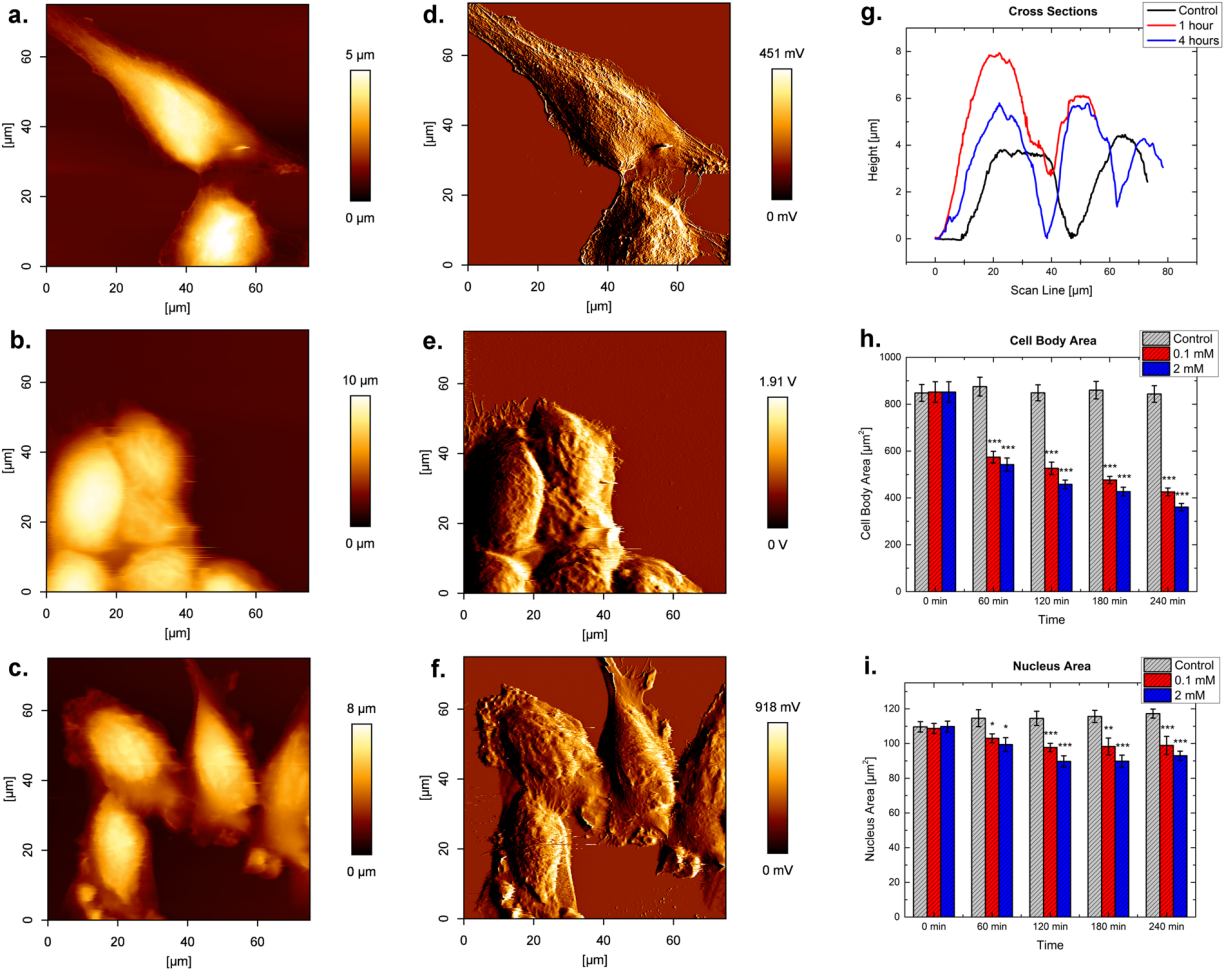
AFM height images in contact mode for control (**a**), cells treated for 1 hour with 2 mM Colchicine (**b**) and for 4 hours (**c**), respectively. (**d**–**f**) Show the corresponding error images. Cells were fixed prior to imaging. The vertical-scale is different for all images. (**g**) Shows respective cross-sections of height images for (**a**–**c**) with the control in black, 1 hour- incubation in red, and 4 hour-incubation in blue. (**h**) Development of the mean cell body area for the control (light grey), 0.1 mM (red) and 2 mM (blue) colchicine over the course of 240 min. (**i**) Development of the nuclear area for the control (light grey), 0.1 mM (red) and 2 mM (blue) colchicine over 240 min. Asterisks indicate the changes with statistical significance.

A combination of optical and surface probing microscopy was used to study changes in cell shape, cell area and cell height due to microtubule depolymerization. AFM contact mode height images of fixed HUVEC cells in PBS for control, one and four hours of incubation with 2 mM colchicine are shown in Fig. 1a–c, while panels d-f depict the respective error images. A contraction of the cell body together with the presence of bleb-like structures near the cell rim were observed. The control cells showed the typical semi-confluent appearance of endothelial cells (see Fig. 1 as well as Figs S1 and S2). Colchicine incubation led to different cell shapes with a higher amount of protrusions. In addition, cell height above the nucleus increased from 4.1 ± 0.1 µm to 6.7 ± 0.3 µm after one-hour exposure, reaching a final value of about 5.1 ± 0.3 µm after 4 hours (Fig. 1g). For untreated cells, filamentous sub-surface structures were visible. Furthermore, the combined effect of contraction and the increase in cellular height led to imaging artifacts (see Supplementary Information, Fig. S3).

Treatment with both 0.1 and 2 mM colchicine produced a significant reduction of the cell area, as studied by calcein fluorescence (membrane staining). After 240 min, the cell area decreased from 840 µm^2^ to 400 µm^2^ (see Fig. 1h and Supplementary Information, S4). Furthermore, microtubule disruption induced a significant decrease in the nucleus area (Fig. 1i), while no conclusive changes in nucleus shape (circularity, aspect ratio) were determined (Supplementary Information, Fig. S5).

### Cytoskeletal rearrangements followed by confocal fluorescence microscopy

**Figure 2:**
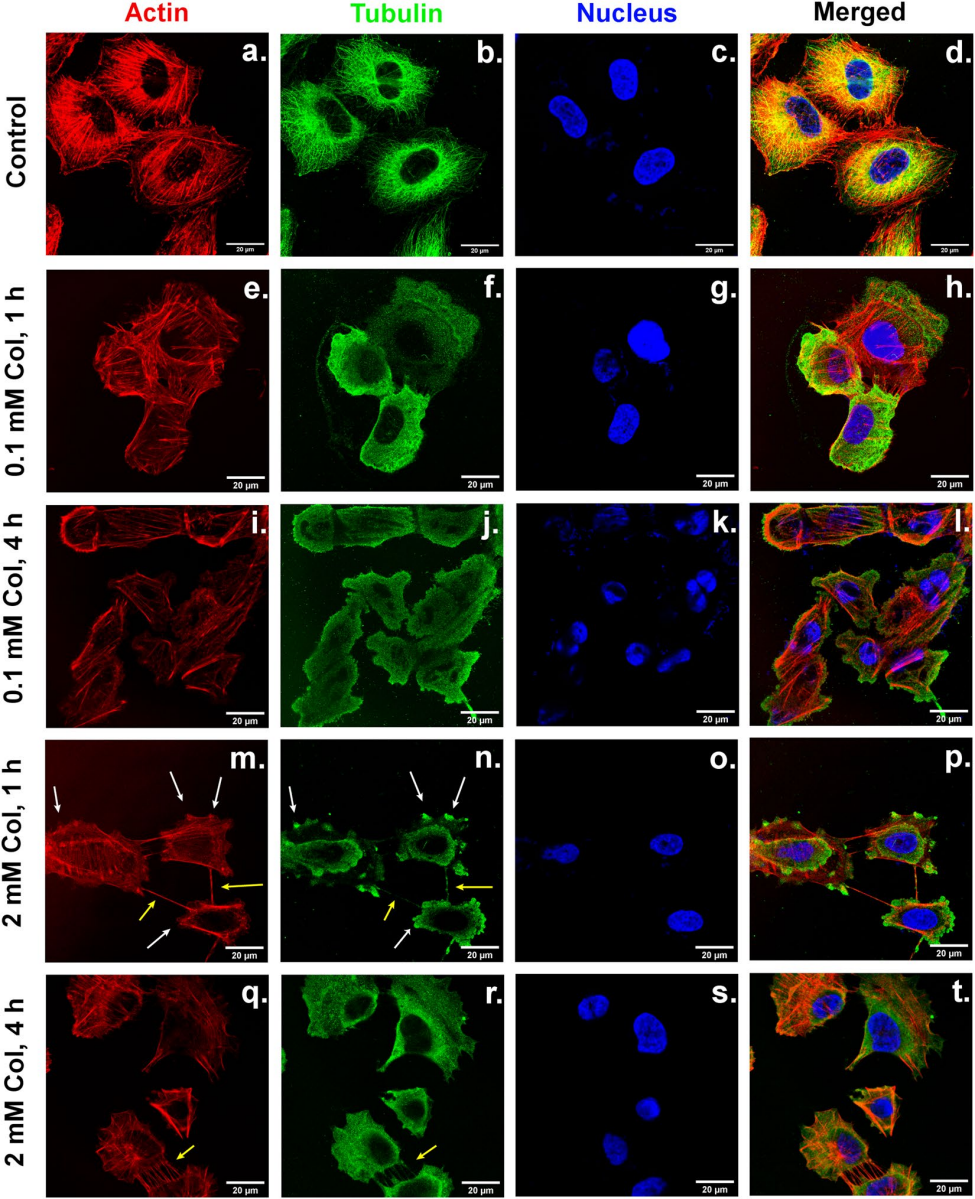
Confocal laser scanning fluorescence microscopy of cells for control, 0.1 mM and 2 mM with actin filaments (red), microtubules (green), nucleus (blue), and a composite image of all stainings. Panels (a–d) show the control cells. Panels (e–h) depict 1 hour of 0.1 mM Colchicine exposure. Panels (i–l) shows 4 hours of 0.1 mM treatment, Panels (m–p) illustrate the effect of 2 mM Colchicine after 1 hour. Panels (q–t) refer to 2 mM Colchicine for four hours treatment. White arrows indicate protrusions while yellow arrows show tethers between cells. The scale bar corresponds to 20 µm.

Confocal fluorescence microscopy was performed to study the rearrangement of the cytoskeleton (microtubules, actin filaments) and the nucleus, as exposure to colchicine took place. This is depicted in Fig. 2, showing control cells (first row, a-d), after one hour (e-h) or four hours (i-l) of incubation with 0.1 mM and after one (m-p) or four hours (q-t) with 2 mM colchicine. The above-mentioned cell area reduction and cell body contraction can be seen again in this figure. The control cells showed a well-defined cytoskeleton with spread tubule fibers from the nucleic site over the whole cell body. A high amount of the actin filaments was present in filamentous form rather than in globular one (the so-called actin stress fibers, important for distributing pressure and forces over the cell body). After one hour of incubation with 0.1 mM colchicine, microtubules were already completely depolymerized. The homogenous distribution of tubulin fluorescence intensity (Fig. 2f) indicates a uniform distribution of the tubulin dimers over the cell body. Actin remained mostly in filamentous form, although the thickness and length of the filaments appeared to be smaller in accordance with cell body shrinkage. The overall cell shape was still similar to the untreated cells, although cell rims were not as well defined. The four-hour treatment produced cells of highly reduced area, more prominent actin stress fibers at the cell edges, and uniformly distributed tubulin dimers. The observed cells presented a higher diversity of shapes in comparison with untreated endothelial cells.

Treatment with 2 mM colchicine changed more abruptly the integrity of the cell cytoskeleton. After one hour, the actin stress fibers were still visible, mostly situated near the cell edge. Protrusions of the cell body appeared in this case, being filled with both actin and tubulin (see white arrows in Fig. 2m,n). For the microtubular network, distinct regions of depolymerized dimer distribution were monitored. The high fluorescence intensity of tubulin in both the protrusions and around the nucleus depict a high tubulin concentration at those places. The cell area was reduced and the cellular shape differed significantly from the shape of the untreated cells. Additionally, the formation of tethers between the cells was recorded. As shown in panel m-n, those structures were filled both with actin and tubulin molecules (seen with yellow arrows). After four hours of colchicine exposure, most of the protrusions disappeared, while some tethers between the cells were still visible (panels q-r). The tubulin concentration seemed to be equally distributed over the cell bodies. No conclusions towards an increase or decrease of the amount of actin present as fibers could be derived from the images shown in Fig. 2. Additional images are found in the Supplementary Information, Figs S6 and S7.

### Cell mechanical properties

**Figure 3:**
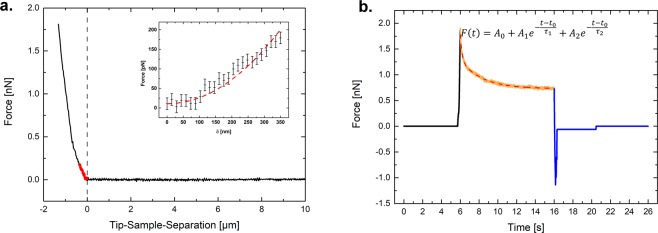
Representative forces curves and mechanical models used in this study. (**a**) Force-Distance curve for the indentation segment. Highlighted in red is the region for which the Hertz model with Sneddon extension is fitted (350 nm). The insert shows the fitting. (**b**) Force-Time curve for a whole measurement, with indentation (black), stress relaxation (orange) and retraction (blue). The fit for the used stress relaxation model is seen as a dashed red line. The equation used is shown in the inset (see the Methods section for more information).

Figure 3 shows the models used for determining cell mechanical properties such as the apparent elastic modulus and the stress relaxation. A Hertz contact model with Sneddon extension for a pyramidal indenter geometry was well suited to fit our data sets with an indentation depth of 350 nm (see Fig. 3a). The insert in Fig. 3a depicts a fitting of Eq. 2. Furthermore, Fig. 3b shows the fitting of the force relaxation curve. A summary of data processing steps can be found in the Supplementary Information, Fig. S8.

#### Apparent Elastic modulus (Young’s Modulus)

**Figure 4:**
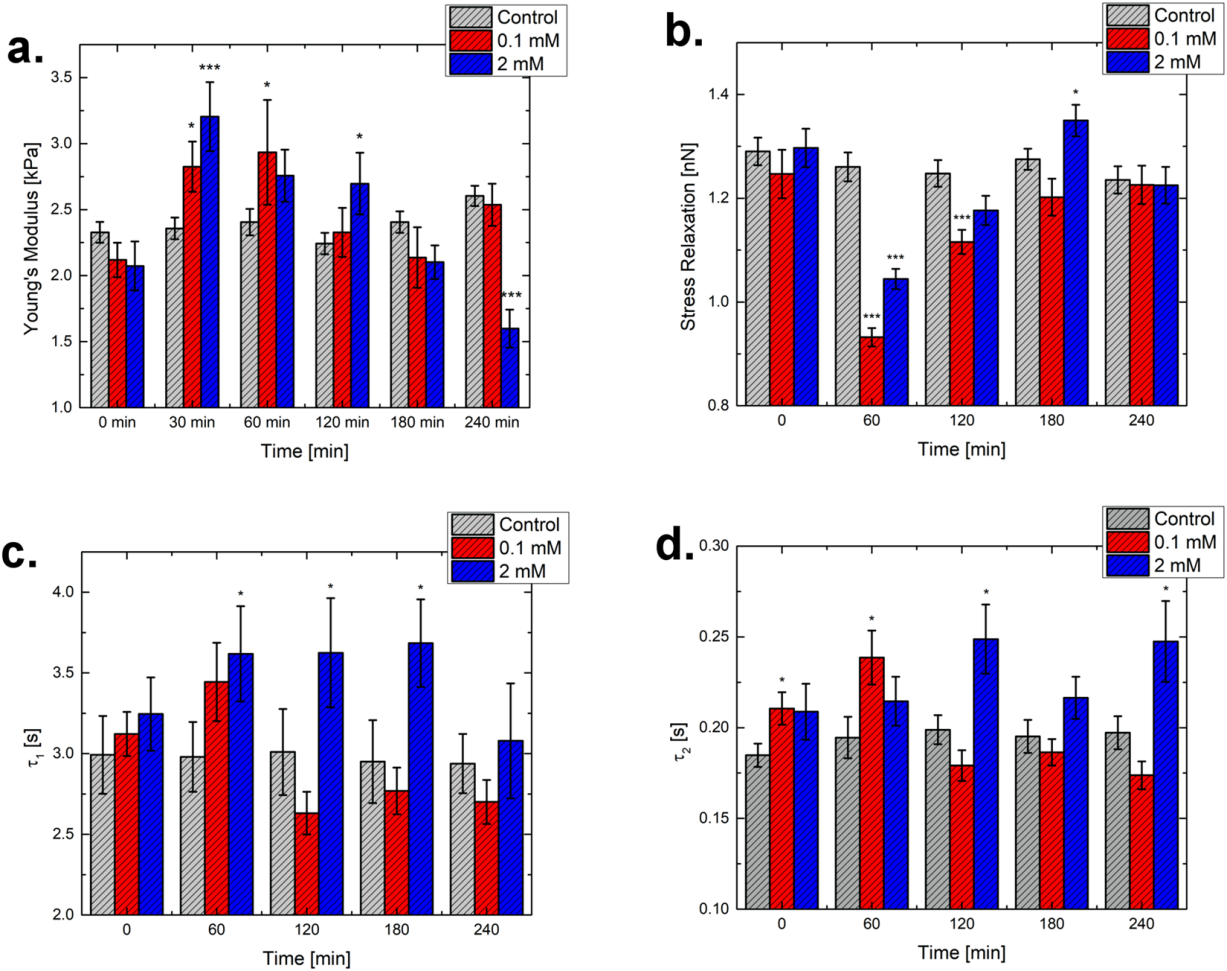
Mechanical properties of cells for control (light grey), 0.1 mM (red) and 2 mM (blue) colchicine treatment. All values are listed as (mean ± standard error of the mean). (**a**) Development of the Young’s Modulus, (**b**) Stress relaxation, (**c**) First relaxation time and (**d**) Second relaxation time. Asterisks indicate changes in statistical significance between the control and the respective value. A more thorough statistical analysis can be found in the Supplementary Information (S9–S12).

The apparent Young’s Modulus was evaluated as a function of time and colchicine concentration (control, 0.1 mM, 2 mM), the results obtained can be seen in Fig. 4a. All data were normally distributed (see Supplementary Information S9). Control cells (untreated) showed a non-significant time-dependent rise from 2.33 to 2.60 kPa. Incubation with 0.1 mM colchicine led to an increase in Young’s modulus after already 30 min, reaching its maximum value at 60 min (2.93 kPa). Then, the modulus decreased until achieving a similar value as the control (for 180 and 240 min).

For 2 mM colchicine the increase in the modulus responded to a similar trend. It peaked at a maximum of 3.2 kPa after 30 min, decreasing with time until a final value of 1.6 kPa (70% of the starting value). The depicted initial increase of cell stiffness is in agreement with studies performed on other cell lines and tissues^6,33,34^.

#### Stress relaxation behavior

The behavior of cells under an applied load at constant height (so-called stress relaxation) was determined. Here we applied a generalized Maxwell model, which was previously used to describe the stress relaxation behavior of MCF-7 cells. In that study, Moreno-Flores *et al*. determined two distinct relaxation times and the non-uniform distribution of these parameters over the cell body, due to the underlying features^31^. A double-exponential decay behavior, which corresponds to N = 2 in Eq. 2 (see the Methods section), was best suited to fit the force-time curves produced in this study (the equation is depicted inside the Fig. 3b).

The fitting of this model is shown in Fig. 3b. This model describes the cell as composed of two different materials of distinct distinguishable relaxation times. Time and concentration dependence of the overall stress relaxation capability (being A_1_ + A_2_) and the relaxation times τ_1_ and τ_2_ (reciprocal of decay parameter) were evaluated. The datasets were normally distributed. All numerical values and a thorough statistical analysis can be found in the Supplementary Information (S10–S12).

The development of stress relaxation capability is shown in Fig. 4b. For the control group, the value varied slightly over time, starting at 1.290 nN and ending up with 1.235 nN (with an associated error of around 0.02 nN). For both 0.1 and 2 mM, an initial significant reduction of the value was seen after already 60 min (minus 25% for 0.1 and minus 20% for 2 mM). Later, the value converged towards the control data. Concerning the relaxation times (see Fig. 4c,d), τ_1_ was always in the range of seconds, while the other (τ_2_) was around a tenth of a second. For the control values, both relaxation times stayed similar over the experiment time range. For the lower concentration, an initial rise of both relaxation times was followed by a decrease. Only the initial increase showed a statistically significant difference with respect to the control. The incubation with 2 mM colchicine led to an increase of the slower incubation time, which reached a plateau at around 3.6 s from 60 to 180 minutes, to be then followed by a decrease, reaching similarity to the control. The faster relaxation time showed a significant increase compared to the control, most prominent after 120 minutes (25% increase).

### Tip-cell surface interaction and rupture forces

#### Maximum adhesive force between tip and sample

**Figure 5:**
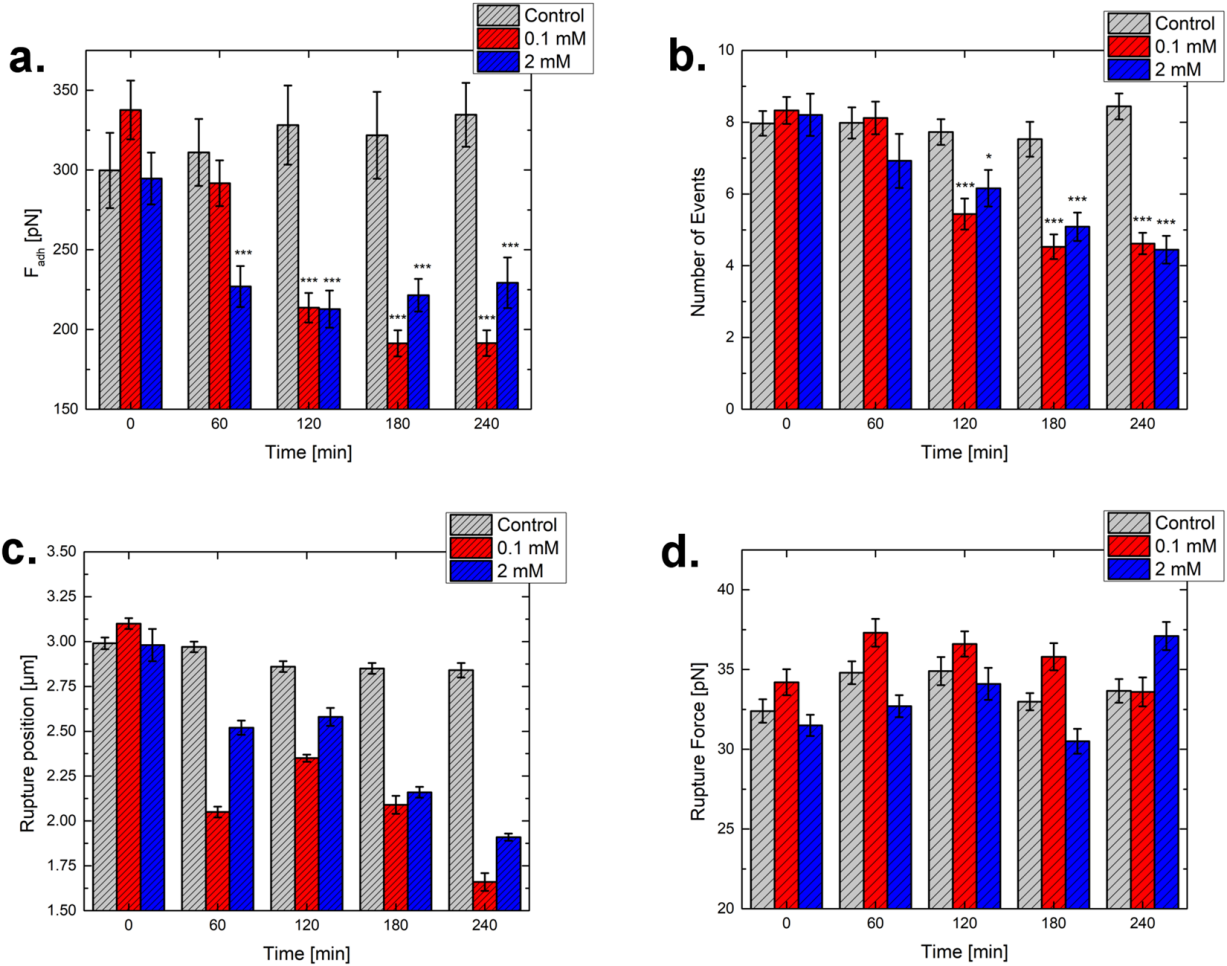
Adhesion between cells and tip for control (light grey), 0.1 mM (red) and 2 mM (blue) colchicine over a time of 240 minutes. (**a**) Shows the maximum cell-tip force (**b**) Number of events per measurement, (**c**) Position of rupture events, and (**d**) Force of the rupture events. For the adhesion force and the number of rupture events, the mean values with the standard error of the mean are shown. For the rupture force and the position, the most probable value gained by extreme value fitting of the value distribution is shown. The statistics can be found in the Supplementary Information (S13, S17 and S18).

Due to the prolonged contact between the cantilever tip and the cell during stress relaxation measurements, manifold attractive interactions form between both (e.g. due to the membrane, membrane proteins). Those interactions are unspecific by their respective nature and are detected by retracting the tip from the cells, therefore showing an adhesive behavior. This can be used to quantify the strength of tip to sample adhesion^35^, by calculating the minimum force value and position of the retract part of the force-distance-curve. Figure 5a shows the development of the maximum adhesive force between tip and cell for control cells and both drug concentrations. While for the control the adhesion increased slightly over time, from 300 pN to 335 pN after 240 min of exposure, a decrease was seen for both colchicine concentrations. For 0.1 mM colchicine, a decrease to a value of 213 pN was recorded after 120 min, while for the high concentration value of around 230 pN was reached already after 60 min. The values then remained quite similar (after 240 min 0.1 mM dropped to 190 pN while 2 mM stayed around 230 pN). In literature, one group evaluated the adhesive work change over the incubation range, reaching quite similar results (a reduction over incubation)^33^. The corresponding statistics can be found in the Supplementary Information (S13).

#### Molecular elasticity events and tether formation

**Figure 6:**
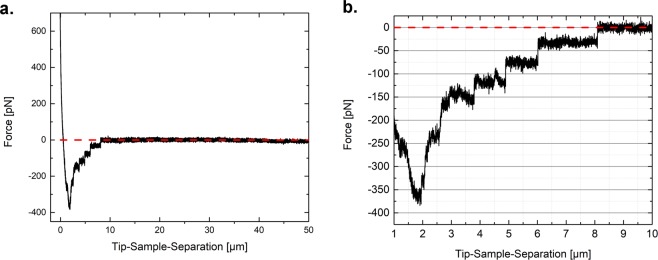
(**a**) A representative example of a retract curve with and adhesion peak and defined step-wise rupture events. The red line indicates the return to zero force (baseline). (**b**) Zoom-in to evaluate rupture events present in the curve.

During retraction of the tip from the cell surface, step-like rupture events were seen in the force-distance-curves. These events appeared at various distances away from the cell, and after the main adhesion peak had been observed (whose manifold interactions involved could not be differentiated by the approach used in this study). Figure 6 shows an example of such behavior. After moving away for some µm, most of the contacts with the cell components were lost, and only so-called membrane tethers were still present between tip and the cell. They showed a characteristic behavior (step-like rupture, force values of around 20 to 100 pN, etc) depending on the cell and the experimental set-up^36,37^. We monitored the number of events per experiment (Fig. 5b), their position (Fig. 5c) and the force needed for rupture (Fig. 5d).

In the Supplementary Information (S14), a comparison of representative retraction curves for different incubation times with 2 mM colchicine can be seen. We noted an apparent difference in rupture event distribution resulting from microtubule disruption and therefore plotted the rupture force of the individual events against the positions (see Supplementary Information S15). This allowed for further characterization of the changes taking place. These data sets were not normally distributed and therefore fitted with an extreme value function, as can be seen in the Supplementary Information S16.

Concerning the number of events per curve, these remained similar for the control (around 8), while for both 0.1 and 2 mM colchicine, a reduction of ca. 50% was seen after 240 min (Fig. 5b). Over the whole incubation period, the most probable position of rupture events stayed around 3 µm for the control, while for 0.1 mM it dropped to around 1.66 µm and for 2 mM to around 1.91 µm (Fig. 5c). No trend was determined for the evolution of the most probable rupture force after microtubule depolymerization (Fig. 5d). Due to the nature of the membrane tether (a hollow cylinder of membrane components), microtubules might not change the properties of the membrane fluidity. The numerical values and statistical analysis can be found in the Supplementary Information (S17 and S18).

## Discussion and Conclusions

This study showed that depolymerization of microtubules by colchicine leads to time and concentration-dependent changes of cell morphological, mechanical and adhesive properties. Using only a pulse-like incubation, no significant changes in cell properties were determined (see Supplementary Information, S19-S26). The reduction in cell area and cell contraction suggests that by the chemical microtubule disruption, the ability of cells to adhere to the surface is decreased. A similar contraction of endothelial cells was reported by various studies^17,38^. The metabolic stress induced by the drug and the influence of its intracellular transport might also lead to changes in cellular adhesion. Interestingly, the behavior was the same for both tested concentrations. These results were supported by the reported cytoskeletal rearrangements. Independent of concentration, the microtubular network is completely disturbed already after one hour of incubation. This led to pronounced changes in cell shape. The cells showed protrusions filled with actin and tubulin. This has a significant impact on cell adhesion, and also on pressure and force distribution over the whole cell body. In addition, it was observed that nuclear shape did not change due to microtubule disruption, as previously reported by Versaevel^39^. In literature, it was reported that incubation with colchicine leads to a change of gene expression in HUVEC cells after exposure of already 30 min^12^. The major genes that were either induced or suppressed by colchicine were those associated with inflammatory processes, neutrophil migration, and the cytoskeleton. Others also reported immediate effects of colchicine on endothelial adhesiveness, changing the quantitative and qualitative display of selectins^40^.

Our results show that the amount of filamentous actin in stress fibers remained approximately similar. However, various studies have reported that the treatment of cells with colchicine induces an alteration of the G/F-actin ratio, which favors actin in its filamentous form by activation of actin polymerization^20,41^. Microtubule disruption leads to the contraction of cells and was often associated with an increase in stress fibers by actin-microtubule crosstalk^18,19^. A possible explanation will be due to changes in phosphorylation of the myosin light chain or RhoA-mediated actin polymerization^38,42^. Since our results did neither fully support nor disprove an increased formation of actin stress fibers, additional studies for quantification of their formation are needed.

A thorough study of elastic (Young’s modulus) and viscoelastic (stress relaxation mechanics, relaxation times) properties was the main body of this work. By definition, Young’s Modulus is a (3-D) deformability property of elastic materials. Due to the viscous nature of cells, it correlates to the instantaneous response of cells to external stress. We reported an initial cell stiffening for both concentrations, followed by an eventual softening. The fast stiffness increase (30 min) could be explained by the formation of actin-myosin stress fibers triggered by microtubule disruption, as reported by other studies^18^. The results from confocal microscopy studies did not clearly show such an effect. A softening of cells treated with colchicine was reported by other studies using a lower concentration but a longer incubation time (at least 14 hours). Here we think that the eventual softening of HUVECs after 240 min with 2 mM colchicine is a similar result^43^. Another study using scanning acoustic microscopy reported quite similar changes in cell mechanics^17^. A theoretical study on cell mechanics after microtubule depolymerization used a tensegrity network approach to describe the different results in the literature (both stiffening and softening reported), concluding that microtubule disruption for cells that are less spread leads to a softening^44^.

The Young’s Modulus and the relaxation time are factors related to the viscosity of the material studied. The changes in the relaxation times appear on the same time-scale as the changes in stiffness, thus indicating that the properties are intertwined, as shown in other works, e.g. for L929 cells^6^. The initial stiffening was accompanied by a slowing down of the relaxation process. In quite a similar study, researchers found a comparable response of a HeLa cell line when disrupting microtubules. They reported an increase in cell height, Young’s Modulus and both relaxation times after treatment with nocodazole for 30 min^33^.

Depending on the technique, cell line and mechanical model used, various relaxation times for cellular structures have been proposed in the literature. Most commonly, research groups reported a (slow) relaxation time in the range of seconds, either employing single or multiple exponential decays and a faster relaxation time in the range of a tenth of a second^45,46^. For instance, Darling *et al*. used a thin-layer stress relaxation model to investigate if metastatic behavior can be determined by measuring cell viscoelasticity^47^. The notion that the fast relaxation time was around a tenth of the slow one is quite interesting and was also reported in other studies^48^. The slow relaxation time is often related to the mechanics of cytoskeleton features. The faster one shows the behavior of the cell membrane (and connected features) under mechanical stress^49^. Concerning colchicine incubation, the increase of both values for intermediate times for the high concentration is thought to again originate from a more distinct organization of the actin filaments, although not entirely visible in the confocal fluorescence experiments. The increase in the fast relaxation time for the high concentration possibly corresponds to a change of the connection of the membrane to the cytoskeleton. A recent publication reported an increase in plasma membrane fluidity due to colchicine incubation^50^.

Processes in cells or in between them occur at different time scales, ranging from nanoseconds for molecular interactions, a few milliseconds for the diffusion of small molecules, seconds for interactions like protein folding and translation, minutes for cellular movement and cytoskeletal rearrangements, to even days considering the cell cycle. In addition, one has to consider the velocity of cellular movement (being in the range of around 10 µm/hour for endothelial cells), a process which is controlled by directed, polarized cytoskeletal rearrangements. A comparison of these numbers with our results indicates that the changes in HUVEC cell mechanics happen on a similar time-scale as cytoskeletal remodeling.

A revision of literature also shows that microtubule disruption leads to significant changes in the expression of genes associated with cellular adhesion in endothelial cells and neutrophils, therefore changing the ability of adhesion^12,40^. These results are partly supported by our findings. Microtubules are an integral part of cell-cell as well as cell-substrate adhesion complex formation. Their disruption leads to alterations in the constitution and properties of various adhesion complexes related to cell-cell and cell-surface interactions^51^. Although in this study no single cell probe force spectroscopy measurements were performed (which indicate “real” changes in adhesive properties), the significant reduction of the maximum adhesive force between the tip and the cell surface as well as the reduction in cell area supports the notion that the cellular adhesion properties diminish.

The reduction of the number of rupture events as well as the events being nearer to the cell (from 3 to 1.5 µm for 0.1 mM and 1.9 µm for 2 mM) might be linked to the changes in membrane and cell surface properties caused by the loss of microtubules^52,53^. For 2 mM, this also fits together with the change of membrane fluidity. Assuming that the membrane tethers are hollow cylinders formed from membrane molecules, the force needed to rupture the connection between tip and sample should not strictly depend on the state of the cytoskeleton, thus explaining the non-visible trend of the rupture force. Other studies have tested the influence of disrupting the actin filament network on the formation of membrane tethers^54^. They reported a reduction of the force needed to tear the tethers, mostly because of the loss of attachment of transmembrane proteins like cadherins and integrins to the F-actin. Similar results were reported by a recent work after disruption of the actin network of a confluent epithelial cell layer with Latrunculin^55^. The role of microtubules on e.g. focal adhesion formation is today quite well studied^56^, which could also be a further explanation of the proposed changes.

This work focused on the role of microtubules in cell mechanics, and showed the complex interplay of cellular components in controlling cell shape, mechanics and adhesive properties. While some of the determined changes in the parameters could be explained, the elucidation of others requires additional experiments. Performing additional force spectroscopy studies with cells as probe (as e.g. done to determine the invasivity of different cancer cell lines^36^) would be a further step in elucidating the role of microtubules. Also, a more complete evaluation of cell mechanics by measuring the creep response seems helpful to determine the viscosity of the different cellular constituents.

## Methods

### Cell Culture and Sample Preparation

Human Umbilical Vein Endothelial Cells (HUVEC) were grown in T75 flasks using high glucose Dulbecco’s Modified Eagle Medium with stable glutamine and methyl red. The medium was supplemented with 10% Fetal Bovine Serum and 1% penicillin/streptomycin. This cell line was chosen because it is a model endothelial cell line and therefore also anchorage-dependent. Cells were cultivated at 37 °C with 5% CO_2_ and 95% relative humidity at a maximum confluence of 80%. Before measurements, cells were trypsinized using 2 mL TrypLE^TM^ Express, centrifuged and counted. For AFM and FM measurements, borosilicate cover glass slides (diameter of 24 mm, thickness of 0.08 to 0.12 mm) were rinsed with EtOH (96%), N_2_ dried and cleaned with oxygen plasma for 20 s. The slides were then incubated for 24 h with 4 × 10^4^ cells/mL, suspended in above-described medium. For confocal fluorescence microscopy studies, cells were treated similarly, but instead, a µ-Slide 8 well plate with a glass bottom was incubated. For all measurements, the medium was changed to Leibovitz’s L-15 medium (CO_2_-independent). Media, cell culture cues and other compounds above were all provided by Thermo Fisher Scientific (USA).

### Disruption of microtubules

To evaluate the impact of microtubules on cell mechanical and adhesive properties, cells were incubated with colchicine. For this, colchicine (Sigma Aldrich, USA) was dissolved in a few droplets of DMSO and diluted with cell culture medium until a stock concentration of 5 mM was reached. For microtubule disruption, incubation of the cells at 0.1 and 2 mM over a period of 4 hours was performed. An additional control, pulsed-like way, was performed by adding 2 mM colchicine to the media for a short period of time (ca. 30 seconds).

### Fluorescence microscopy

Fluorescence microscopy studies on the cell area were performed using a Nikon Eclipse TE2000-S (Nikon Instruments, Japan) inverse fluorescence microscope with a LH-M100C mercury lamp and GFP (G) bandpass filter (exc. 480, em. 535 nm). A 20x air objective and a 10x were used. Staining of the cell body was done with Calcein AM (1:100) (Sigma Aldrich, USA). Staining of the nucleus was performed using Hoechst dye (Sigma Aldrich, USA). Micrographs were processed with the Fiji distribution of ImageJ and the ZEN Imaging Software (Zeiss). The cell area of at least three independent samples was measured for all cells in the area of the micrograph at 20 °C.

### Cell fixation, permeabilization, and immunostaining/staining

To evaluate the impact of microtubule depolymerization on the cytoskeleton, confocal laser scanning microscopy experiments were performed, with prior staining of nucleus, microtubules and actin filaments. 5 × 10^4^ cells/mL were seeded on a µ-Slide 8 well plate (Ibidi, Germany) and incubated overnight. Cells were fixed with 4% paraformalydehyde for 10 min at 37 °C. Then cells were permeabilized using 0.1% Triton X-100 (diluted in PBS) at 20 °C for 15 min. They were then blocked using 2% BSA (in PBS) at 4 °C overnight. For immunostaining, primary anti-α-Tubulin antibody diluted in 1% BSA in PBS was incubated at room temperature for 3 hours. Then the secondary antibody (in this case goat anti-mouse IgG, diluted in 1% BSA) with Alexa Fluor 488 was added and incubated for 45 min together with fluorescently labeled phalloidin (Alexa Fluor 555), to stain the actin filaments (diluted in 1% BSA in PBS). Finally, the nuclei were stained using Hoechst (diluted in 1% BSA in PBS). Samples were kept at 4 °C and measured as soon as possible. At least 2 independent samples (with the respective controls) were produced. All materials were purchased from Thermo Fisher Scientific (USA).

### Confocal laser scanning fluorescence microscopy

Confocal scanning microscopy studies were performed on a Leica SP8, equipped with various detectors (2 HyDs, 2 PMTs), a 405 nm and a tunable white light laser (tunable from 470 to 670 nm). Measurements were performed using a 63x oil immersion objective (RI of 1.4) in L-15 media at room temperature. Only single optical sections were recorded with a resolution of 1024 × 1024 pixels (116 × 116 µm). Micrographs were later processed using Fiji distribution of ImageJ.

### Atomic force microscopy

Measurements were performed on a JPK Nanowizard III (JPK Instruments, Germany) with a CellHesion module mounted on an inverted optical microscope (Axio Observer Z1, Zeiss) at 37 °C. Triangular, untreated silicon nitride cantilevers with four-sided pyramidal tips and nominal spring constant of 0.12 N m^−1^ were used (Bruker, DNP-S, cantilever B). Spring constant calibration was performed using the thermal noise method^57^. The cells were approached at a loading rate of 5 µm s^−1^ with a maximum force set-point of 2 nN. The tip was then held at a constant, fixed height above the cell for 10 s (stress relaxation measurement). After that, the tip was retracted at 5 µm s^−1^ and its motion was recorded for 50 µm. The loading rate was chosen after preliminary measurements as described in literature^58^. No membrane ruptures were observed during indentation tests with 1 and 5 µm s^−1^, thus the latter was chosen. The force set-point of 2 nN was chosen to probe also cytoskeletal features during stress relaxation (ensuring deep enough indentations). Each cell was indented at least 10 times with at least 10 cells being probed per time step. Between the indentation of cells, the substrate was probed to ensure tip cleanliness. Cells were indented above the nucleus region to reduce variability and substrate artifacts.

### Atomic force microscopy imaging

For AFM imaging, HUVEC cells were fixed in 4% paraformaldehyde after the respective incubation (normal media, 1 hour or 4 hours of 2 mM Colchicine). The measurements were performed in contact mode with a force set-point between 0.4 and 0.8 nN, a line scan rate of 0.2 to 0.5 Hz, and scan sizes of either 75 × 75 or 100 × 100 µm. Feedback values, as well as scan rates and applied force, were optimized for each image individually. A triangular MLCT cantilever (Bruker) with a nominal spring constant of 0.01 N/m, a resonance frequency of 7 kHz (around 1 kHz in liquid) and a tip radius of 20 nm was used. The cantilever was calibrated prior to measurements using the thermal noise method. Measurements were performed in PBS at room temperature and analyzed using the manufacturer software. For cell height determination the average value of fifteen cells per incubation step was calculated.

### Data analysis and fast, automatized calculation

The measured value of the piezo position and the cantilever deflection can be used to determine the indentation, following Hooke’s law, as discussed recently^58^. The R afmToolkit developed by Benitez *et al*. was subsequently used for batch-processing purposes to evaluate the contact point of measurements as well as the Young’s Modulus, adhesion properties and stress relaxation behavior^59^. Usage of this tool allowed fast data evaluation processes, enabling also to calculate additional parameters. The parameters for the algorithm for contact point determination was optimized for all curves. For calculation of *E*, the Sneddon extension of the Hertz model for four-sided pyramidal indenters (Eq. 4) was used

1 $$F=\frac{E}{1-\upsilon }\frac{\tan (\alpha )}{\sqrt{2}}{\delta }^{2}$$

where, E is the Young’s Modulus, ν is the Poisson’s ratio (set to 0.5 for the cells being incompressible), α is the face angle of the pyramid, and δ is the indentation. An indentation of 350 nm (corresponding to a force of around 200 pN) was used to calculate the Young’s Modulus. A typical curve of the approach part of an AFM force-distance measurement as well as the fitting of the model with the R afmToolkit over an indentation of 350 nm is shown in Fig. 6a. For small indentations (below 10% of the material height, cell height was averaged around 5 µm), the assumptions of the model are accomplished.

Evaluation of relaxation mechanics was performed by using a generalized Maxwell model (multi-exponential force decay behavior). This model consists of a parallel arrangement of multiple viscoelastic components. The force decay is then described by

2 $$F(t)=A(0)+\mathop{\sum }\limits_{i=1}^{N}{A}_{i}{e}^{-\frac{(t-{t}_{0})}{{\tau }_{i}}}$$

where A (0) is an instantaneous response, A_i_ the decay amplitude and τ_i_ the respective relaxation time of the individual viscoelastic components. Various exponential decay functions were fitted simultaneously to the data (single, double, triple …) to optimize the fitting of the data. A multi-exponential decay function would mean that the cells under study have different relaxation times which should correspond to different cell constituents. The fitting was performed using the Levenberg-Marquardt non-linear least square method with an optimized self-start model.

### Statistical analysis

Statistical and mathematical analysis was performed using OriginPro 9 (OriginLab Corporation) and R. Normally distributed data sets were evaluated by Gaussian fitting, calculation of mean value and the standard error of the mean. Data not normally distributed around the mean value was fitted using an extreme value function. Paired student t-tests were performed (and, if not feasible, Paired-Sample Wilcoxon Signed-Rank test as a non-parametric test) with a p-value < 0.05 deemed significant (“*” < 0.05, “**” < 0.01, “***” < 0.001).

## Supplementary Information

Supplementary Information
