# Supplementary material for: Microtubule disruption changes endothelial cell mechanics and adhesion
Source: Sci Rep. 2019 Oct 17;9:14903. doi: 10.1038/s41598-019-51024-z (PMC6797797; doi:10.1038/s41598-019-51024-z)
Supplement: Supplementary file 1 — Supplementary Information [file 41598_2019_51024_MOESM1_ESM.pdf]

Microtubule disruption changes endothelial cell mechanics and adhesion

Supplementary Information

Authors: Andreas Weber[1]*, Jagoba Iturri[1], Rafael Benitez[2], Spela Zemljic-Jokhadar[3], José L. Toca-Herrera[1]*

[1] Institute for Biophysics, Department of Nanobiotechnology, University of Natural Resources and Life Sciences Vienna, Muthgasse 11, A-1190 Vienna, Austria. andreas.weber@boku.ac.at, jagoba.iturri@boku.ac.at, jose.toca-herrera@boku.ac.at

[2] Dpto. Matemáticas para la Economía y la Empresa, Facultad de Economía, Universidad de Valencia, Avda. Tarongers s/n, 46022 Valencia, Spain. rafael.suarez@uv.es

[3] Department of Biophysics, Medicine Faculty, University of Ljubljana, Vrazov trg 2, 1000 Ljubljana, Slovenia. spela.zemljic-jokhadar@mf.uni-lj.si

Corresponding author

Andreas Weber: andreas.weber@boku.ac.at
José L. Toca-Herrera: jose.toca-herrera@boku.ac.at

S1 – Micrographs of cells

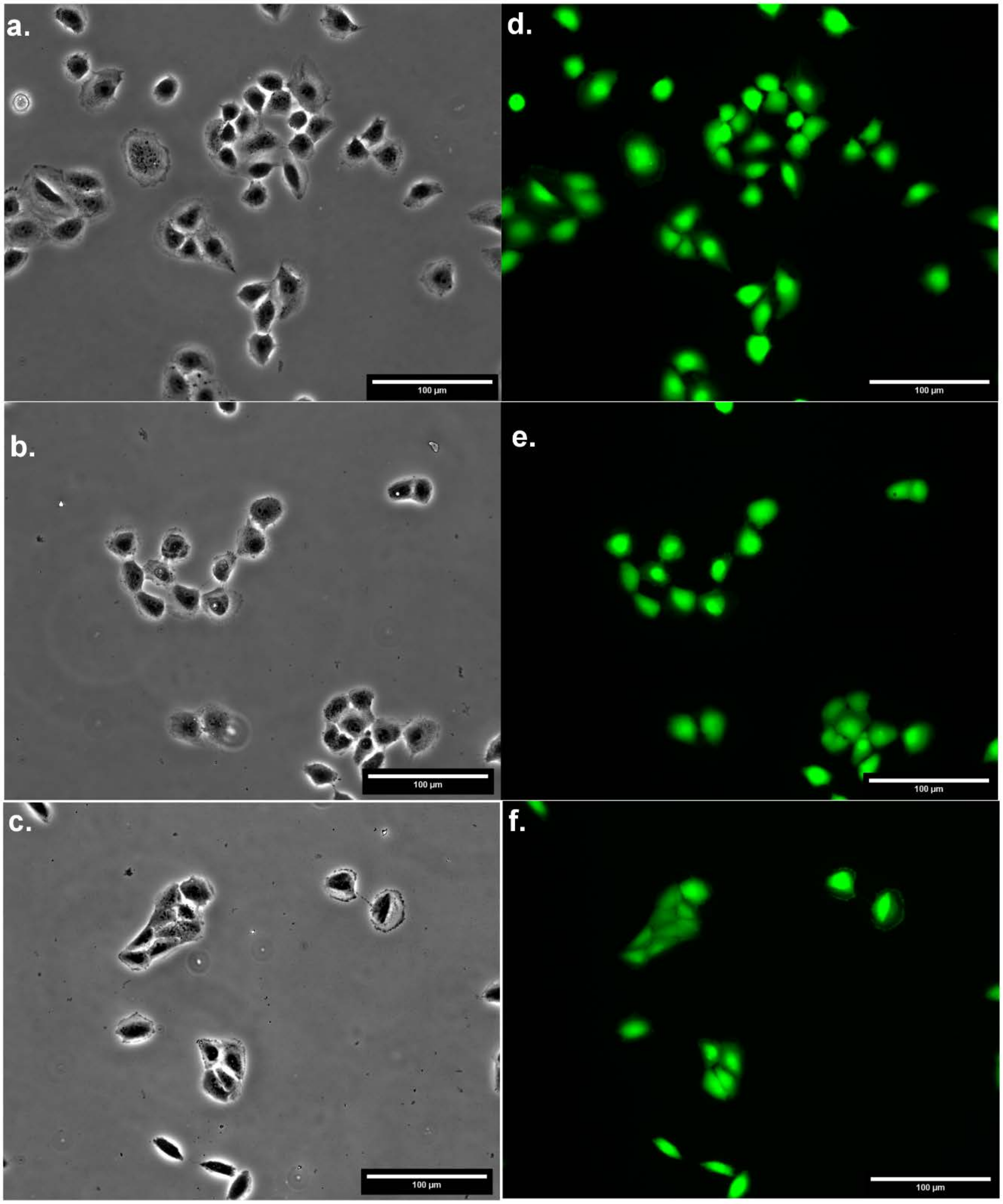

Figure S1. Micrographs (a)-(c) and fluorescence micrographs (d)-(f) of cells, untreated (first row), treated for 1 hour with 2 mM Colchicine (second row) or for 4 hours (third row). Notice the reduction of cell area, an apparent contraction of the cell body and changes in overall cell shape.

S2 – Additional AFM Images

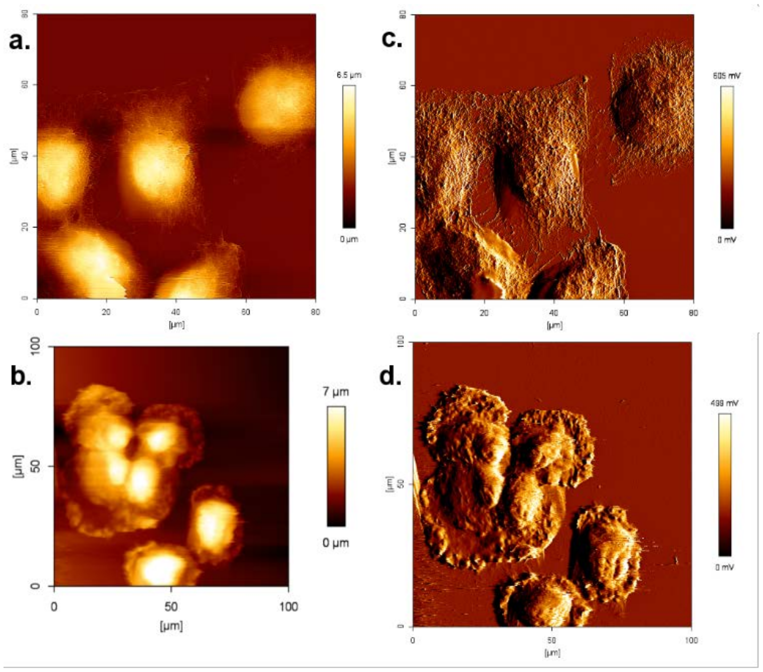

Figure S2. AFM images in contact mode, for untreated cells showing height (a) and error (c), and for 4 hour of 2 mM colchicine treatment, showing height (b) and error (d).

S3 – Artefacts in AFM imaging for 1-hour incubation with 2 mM Colchicine

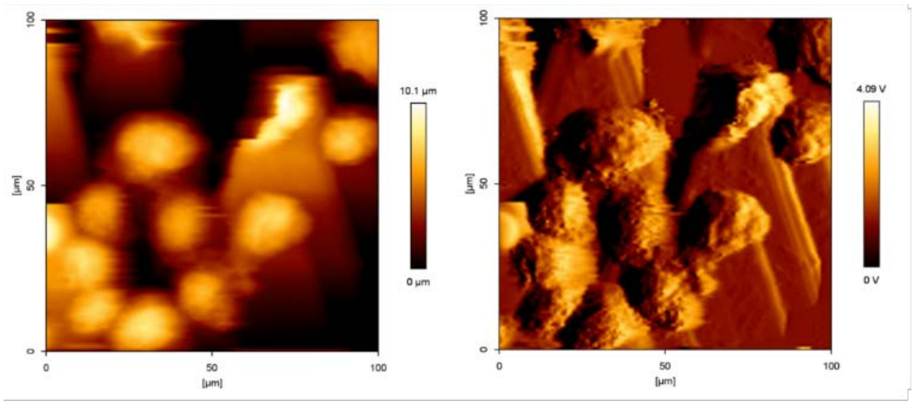

Figure S3. AFM imaging artefacts, for height (left) and error (right) image. This artefact is a result of the cantilever tip (2.5 to 8 µm according to manufacturer) being not as high as the feature changes in cell topography, thus the cantilever body touches the cell, which leads to a bending and can be seen as a "print" of the cantilever geometry in the image (note the triangular shapes in the image). This happened for nearly all cells treated for 1 hour.

S4. Numerical values of cell area and statistical analysis.

Table S4 - 1. Values for cell area (in μm^2), mean value ± standard error of mean.

	Control	0.1 mM	2 mM
**0**	845.0 ± 36.0	851.6 ± 44.0	848.0 ± 44.0
**30**	901.6 ± 33.2	687,6 ± 44.0	655.22 ± 28.0
**60**	874-8 ± 40-8	574.0 ± 24.8	542.0 ± 30.4
**120**	848.4 ± 34.0	526.4 ± 26.4	456 ± 18.4
**180**	860.0 ± 37.2	476,4 ± 15.6	426.8 ± 30.8
**240**	843.6 ± 35.2	413.2 ± 16.4	360.8 ± 15.4

Table S4 – 2. Statistical analysis of value evolution over time.

Control	Control	0.1 mM	0.1 mM	2 mM	2 mM
**0**		**0**		**0**	
**30**	n.s.	**30**	*	**30**	***
**60**	n.s.	**60**	*	**60**	**
**120**	n.s.	**120**	n.s.	**120**	*
**180**	n.s.	**180**	n.s.	**180**	n.s.
**240**	n.s.	**240**	*	**240**	**

Table S4 - 3. Statistical analysis of values over each other.

Control	0.1 mM	Control	2 mM	0.1 mM	2 mM
**0**	n.s.	**0**	n.s.	**0**	n.s
**30**	***	**30**	***	**30**	n.s.
**60**	***	**60**	***	**60**	n.s.
**120**	***	**120**	***	**120**	*
**180**	***	**180**	***	**180**	*
**240**	***	**240**	***	**240**	**

S5 – Nuclear shape development

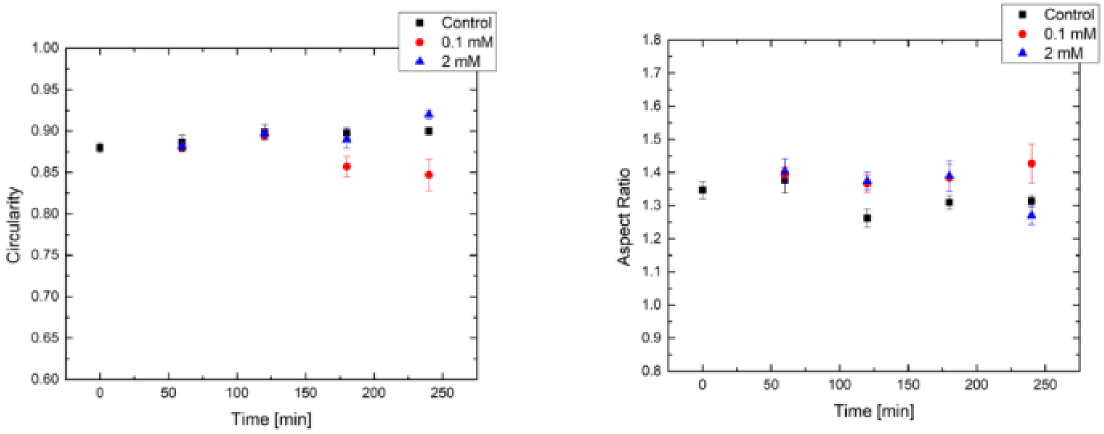

Figure S5. Nuclear shape development, shown as Circularity (left) and Aspect Ratio (right), for control (black), 0.1 mM Colchicine (red) and 2 mM (blue). Circularity was calculated as 4*pi*A/perimeter and aspect ratio as the ratio of both axes.

S6. Time series of 0.1 mM colchicine.

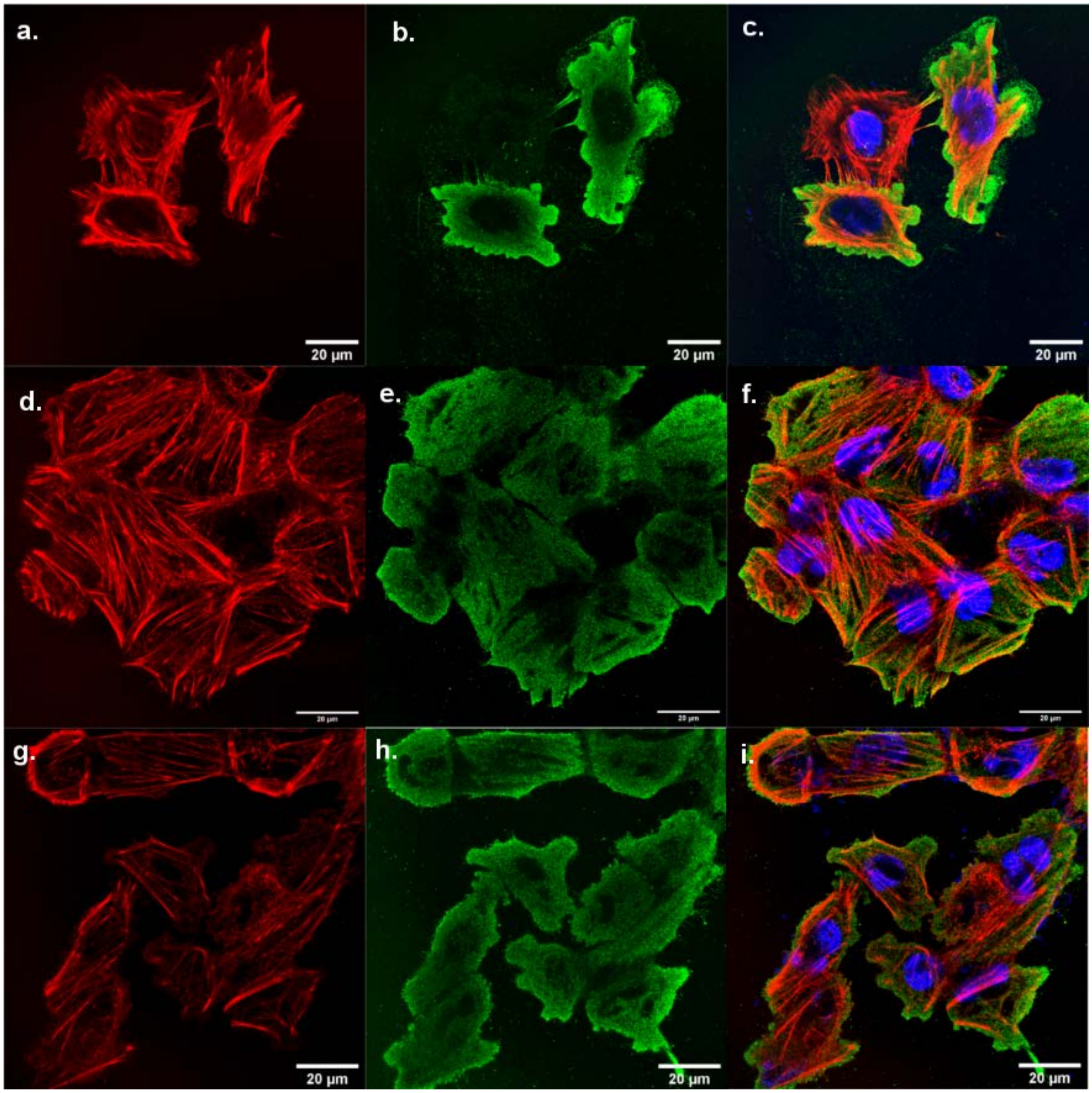

Figure S6. Time series of 0.1 mM colchicine, confocal laser scanning fluorescence. The panels a., d. and g. show actin filaments in red, the panels b., e. and h. microtubules in green, and the panels c., f. and I. a composite (with the nucleus in blue). The first row is after 1 hour of incubation, the second after 2 hours and the third after 4 hours of incubation. Already after 1 hour, cellular shape has changed significantly in comparison to untreated cells. Formation of actin stress fibers is visible, especially after two hours. Already after 1 hour, microtubules seem to be completely depolymerized. Microtubule dimers seem to be distributed evenly over the whole cell body.

S7. Time series of 2 mM colchicine.

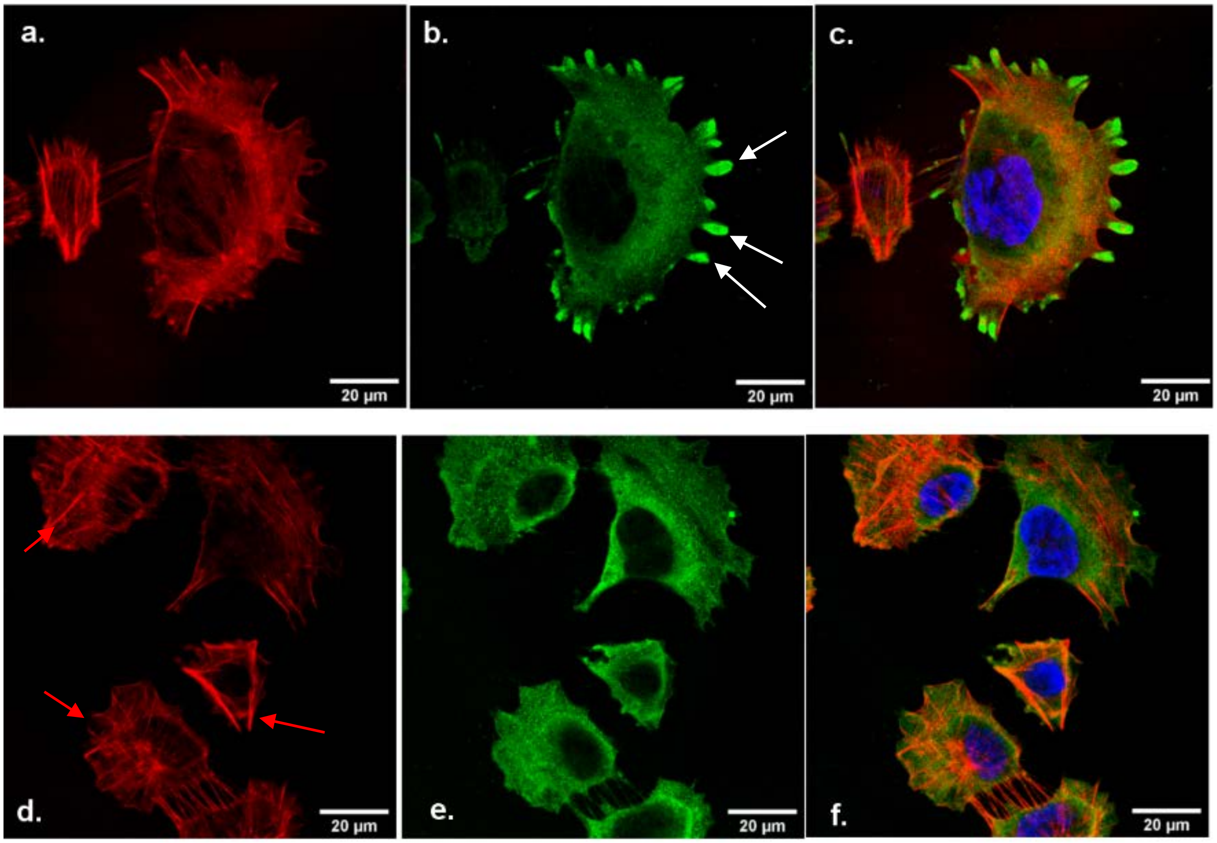

Figure S7. Time series of 2 mM colchicine, confocal laser scanning fluorescence. The panels a. and d. show actin filaments in red, the panels b. and e. microtubules in green, and the panels c. and f. composite (with the nucleus in blue). The first row is after 2 hour of incubation, the second after 4. After 2 hours, microtubules are completely depolymerized. A higher microtubule fluorescence intensity in cell protrusions is visible (indicated by white error in panel b). Actin stress fibers are visible, as indicated by bright red arrows in panel d. Cell shape is highly irregular and diverse.

S8 – Summary of AFM Force Spectroscopy Processing and derived data

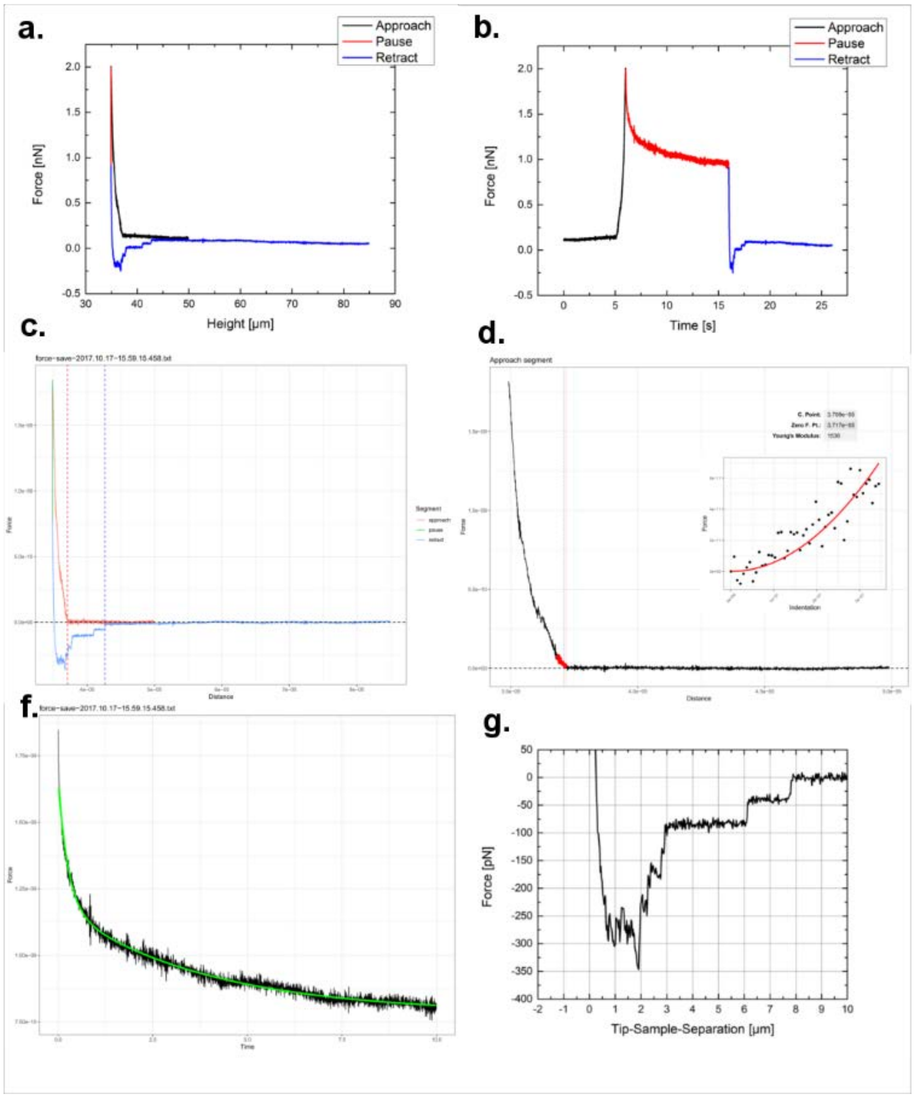

Figure S8. Determination of cell mechanical properties from AFM force spectroscopy measurements using the R afmToolkit. (a) Uncorrected Force-Distance-Curve. (b) Uncorrected Force-Time-Curve. (c) Baseline correction using AFM Toolkit. (d) Fitting of Hertzian mechanics model with Sneddon indentation. (e) Fitting of Stress relaxation model. (f) Evaluation of adhesion pattern und step-like rupture events.

Values derived from this plot: YM 1536 Pa, A0 = 7.83 E-10, A1 = 4.48E-10, A2 = 3.99E-10, tau1 = 3.83 s, tau2 = 0.26 s, Fadh = 348.2 pN. Number of Steps: 7. Position of Steps [µm]: 1.81, 2.15, 2.28, 2.69, 2.82, 6.02, 7.73. Rupture Forces [pN]: 68.7, 56.6, 39.5, 51.8, 46.1, 36.9, 26.3.

S9. Numerical values of Young's Modulus and statistical analysis.

Table S9 - 1. Young's Modulus development (in kPa), mean value with standard error of mean for Control, 0.1 and 2 mM incubation with colchicine.

	Control	0.1 mM	2 mM
**0**	2.33 ± 0.08	2.12 ± 0.13	2.07 ± 0.18
**30**	2.36 ± 0.10	2.82 ± 0.19	3.20 ± 0.26
**60**	2.40 ± 0.08	2.93 ± 0.40	2.76 ± 0.20
**120**	2.24 ± 0.09	2.33 ± 0.19	2.67 ± 0.23
**180**	2.40 ±0.08	2.14 ± 0.23	2.10 ± 0.13
**240**	2.60 ± 0.08	2.54 ± 0.16	1.60 ± 0.14

Table S9 – 2. Statistical analysis of value evolution over time.

Control	Control	0.1 mM	0.1 mM	2 mM	2 mM
**0**		**0**		**0**	
**30**	n.s.	**30**	***	**30**	***
**60**	n.s.	**60**	n.s.	**60**	n.s.
**120**	n.s.	**120**	n.s.	**120**	n.s.
**180**	n.s.	**180**	n.s.	**180**	*
**240**	n.s.	**240**	n.s.	**240**	*

Table S9 - 3. Statistical analysis of values over each other.

Control	0.1 mM	Control	2 mM	0.1 mM	2 mM
**0**	n.s.	**0**	n.s.	**0**	n.s.
**30**	*	**30**	***	**30**	n.s.
**60**	*	**60**	n.s.	**60**	n.s.
**120**	n.s.	**120**	*	**120**	n.s.
**180**	n.s.	**180**	n.s.	**180**	n.s.
**240**	n.s.	**240**	***	**240**	***

S10. Stress relaxation, numerical values and statistical analysis

Table S10 - 1. Stress relaxation development (in nN), mean value with standard error of mean for Control, 0.1 and 2 mM incubation with colchicine.

	Control	0.1 mM	2 mM
**0**	1.29 ± 0.03	1.25 ± 0.05	1.23 ± 0.04
**60**	1.26 ± 0.03	0.93 ± 0.02	1.04 ± 0.02
**120**	1.25 ± 0.03	1.12 ± 0.02	1.18 ± 0.03
**180**	1.27 ± 0.02	1.20 ± 0.04	1.35 ± 0.03
**240**	1.23 ± 0.03	1.23 ± 0.04	1.22 ± 0.03

Table S10 – 2. Statistical analysis of value evolution over time.

Control	Control	0.1 mM	0.1 mM	2 mM	2 mM
**0**		**0**		**0**	
**60**	n.s.	**60**	***	**60**	***
**120**	n.s.	**120**	***	**120**	***
**180**	n.s.	**180**	*	**180**	***
**240**	n.s.	**240**	n.s.	**240**	*

Table S10 - 3. Statistical analysis of values over each other.

Control	0.1 mM	Control	2 mM	0.1 mM	2 mM
**0**	n.s.	**0**	n.s.	**0**	n.s.
**60**	***	**60**	***	**60**	*
**120**	***	**120**	n.s.	**120**	n.s.
**180**	n.s.	**180**	*	**180**	**
**240**	n.s.	**240**	n.s.	**240**	n.s.

S11. τ_1, numerical values and statistical analysis

Table S11 - 1. τ_1 development (in s), mean value with standard error of mean for Control, 0.1 and 2 mM incubation with colchicine.

	Control	0.1 mM	2 mM
**0**	2.99 ± 0.24	3.12 ± 0.14	3.25 ± 0.23
**60**	2.98 ± 0.22	3.44 ± 0.24	3.62 ± 0.29
**120**	3.01 ± 0.27	2.63 ± 0.13	3.62 ± 0.34
**180**	2.95 ± 0.26	2.77 ± 0.14	3.68 ± 0.27
**240**	2.94 ± 0.18	2.70 ± 0.14	3.08 ± 0.36

Table S11 – 2. Statistical analysis of value evolution over time.

Control	Control	0.1 mM	0.1 mM	2 mM	2 mM
**0**		0		0	
**60**	n.s.	60	n.s.	60	n.s.
**120**	n.s.	120	**	120	n.s.
**180**	n.s.	180	n.s.	180	n.s.
**240**	n.s.	240	n.s.	240	n.s.

Table S11 - 3. Statistical analysis of values over each other.

Control	0.1 mM	Control	2 mM	0.1 mM	2 mM
**0**	n.s.	**0**	n.s.	**0**	n.s.
**60**	n.s.	**60**	*	**60**	n.s.
**120**	n.s.	**120**	*	**120**	*
**180**	n.s.	**180**	*	**180**	*
**240**	n.s.	**240**	n.s.	**240**	n.s.

S12. τ_2, numerical values and statistical analysis

Table S12 - 1. τ_2 development (in s), mean value with standard error of mean for Control, 0.1 and 2 mM incubation with colchicine.

	Control	0.1 mM	2 mM
**0**	0.18 ± 0.01	0.21 ± 0.01	0.21 ± 0.01
**60**	0.19 ± 0.01	0.24 ± 0.01	0.21 ± 0.01
**120**	0.20 ± 0.01	0.18 ± 0.01	0.25 ± 0.02
**180**	0.20 ± 0.01	0.19 ± 0.01	0.22 ± 0.01
**240**	0.20 ± 0.01	0.17 ± 0.01	0.25 ± 0.02

Table S12 – 2. Statistical analysis of value evolution over time.

Control	Control	0.1 mM	0.1 mM	2 mM	2 mM
**0**		0		0	
**60**	n.s.	60	n.s.	60	n.s.
**120**	n.s.	120	***	120	n.s.
**180**	n.s.	180	n.s.	180	n.s.
**240**	n.s.	240	n.s.	240	n.s.

Table S12 - 3. Statistical analysis of values over each other.

Control	0.1 mM	Control	2 mM	0.1 mM	2 mM
**0**	*	**0**	n.s.	**0**	n.s.
**60**	*	**60**	n.s.	**60**	*
**120**	n.s.	**120**	*	**120**	*
**180**	n.s.	**180**	n.s.	**180**	n.s.
**240**	n.s.	**240**	*	**240**	**

S13. Maximum adhesive force, numerical values and statistical analysis

Table S13 - 1. Maximum adhesive force development (in pN), mean value with standard error of mean for Control, 0.1 and 2 mM incubation with colchicine.

	Control	0.1 mM	2 mM
**0**	299.7 ± 23.6	337.6 ± 18.4	294.7 ± 16.2
**60**	311.0 ± 20.9	291.7 ± 14.3	227.0 ± 12.9
**120**	328.2 ± 24.8	213.7 ± 9.2	212.8 ± 11.6
**180**	321.8 ± 27.2	191.3 ± 8.2	221.5 ± 10.2
**240**	334.6 ± 20.0	191.5 ± 8.1	229.3 ± 15.9

Table S13 – 2. Statistical analysis of value evolution over time.

Control	Control	0.1 mM	0.1 mM	2 mM	2 mM
**0**		0		0	
**60**	n.s.	60	*	60	**
**120**	n.s.	120	***	120	n.s.
**180**	n.s.	180	n.s.	180	n.s.
**240**	n.s.	240	n.s.	240	n.s.

Table S13 - 3. Statistical analysis of values over each other.

Control	0.1 mM	Control	2 mM	0.1 mM	2 mM
**0**	n.s.	**0**	n.s.	**0**	n.s.
**60**	n.s.	**60**	***	**60**	n.s.
**120**	**	**120**	***	**120**	***
**180**	***	**180**	***	**180**	*
**240**	***	**240**	***	**240**	*

S14. Comparison of retract curves for different incubation times for 2 mM colchicine.

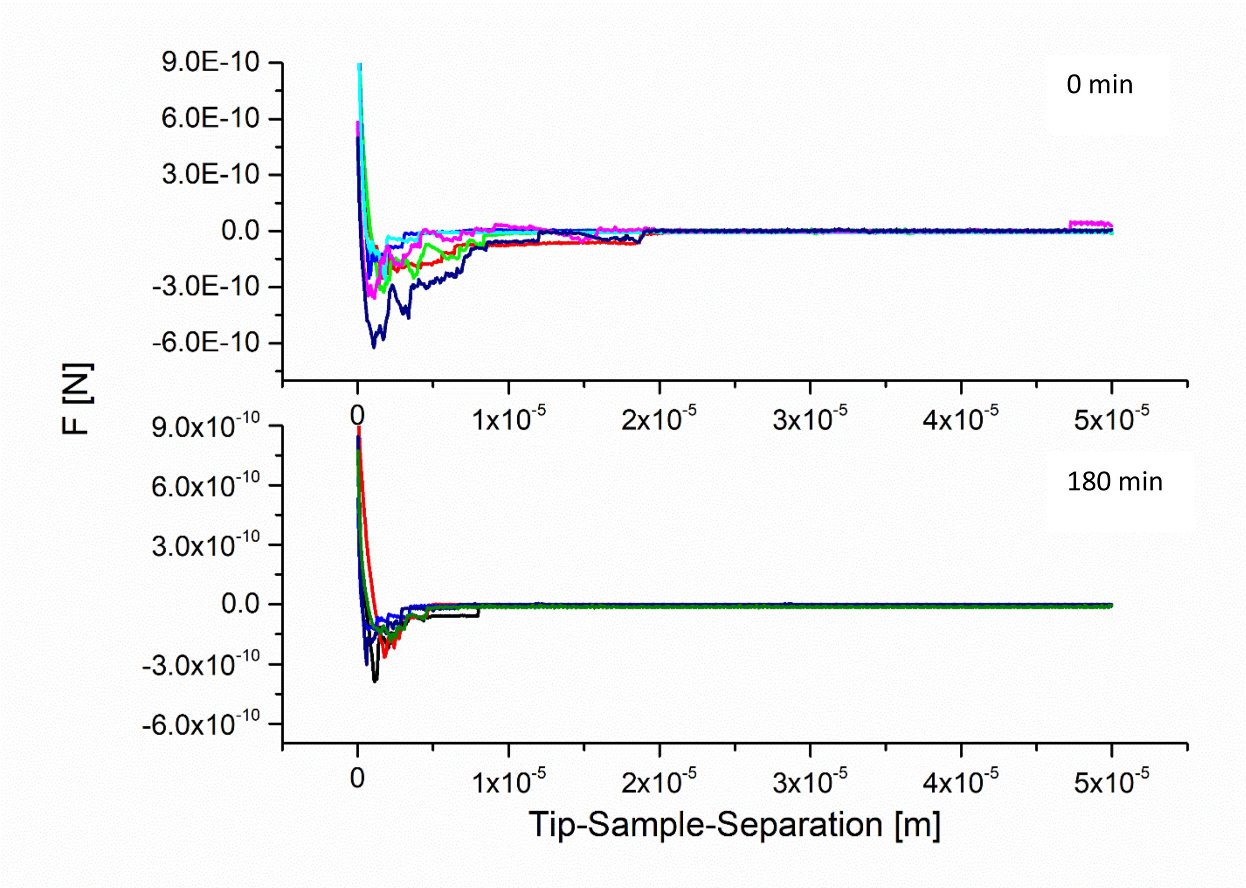

Figure S14. Comparison of adhesion curves (retract curves) for 2 mM colchicine incubation, after 0 min (control, upper panel) and after 180 min (lower panel). Qualitative comparison of the curves shows that the adhesion pattern differs for different incubation times.

S15. Comparison rupture event distribution for different incubation times for 2 mM colchicine.

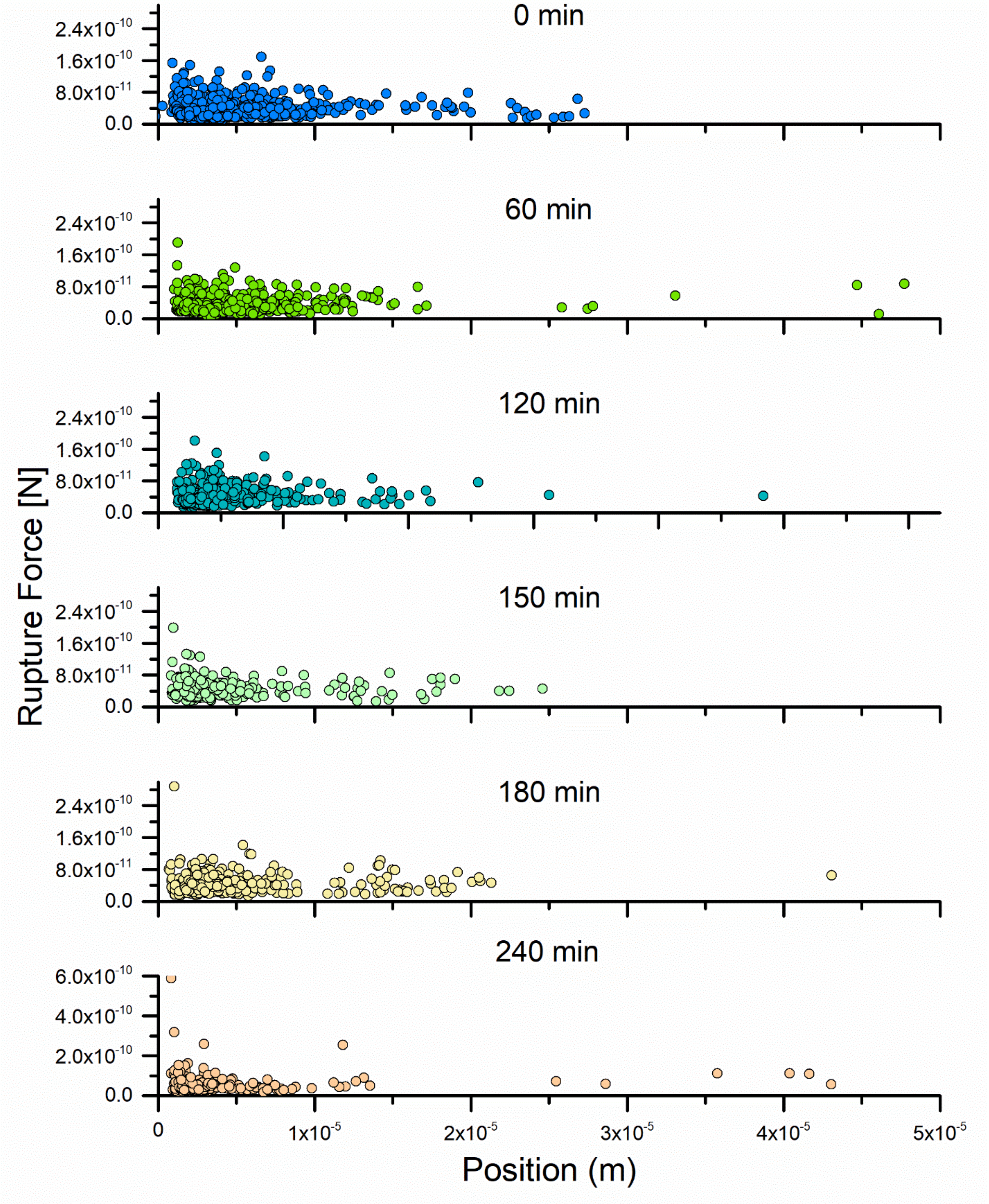

Figure S50. Comparison of Rupture events for different incubation times (from 0 to 240 min) for an incubation with 2 mM colchicine. A change of the distributions is visible.

S16. Extreme value fitting of rupture force distribution.

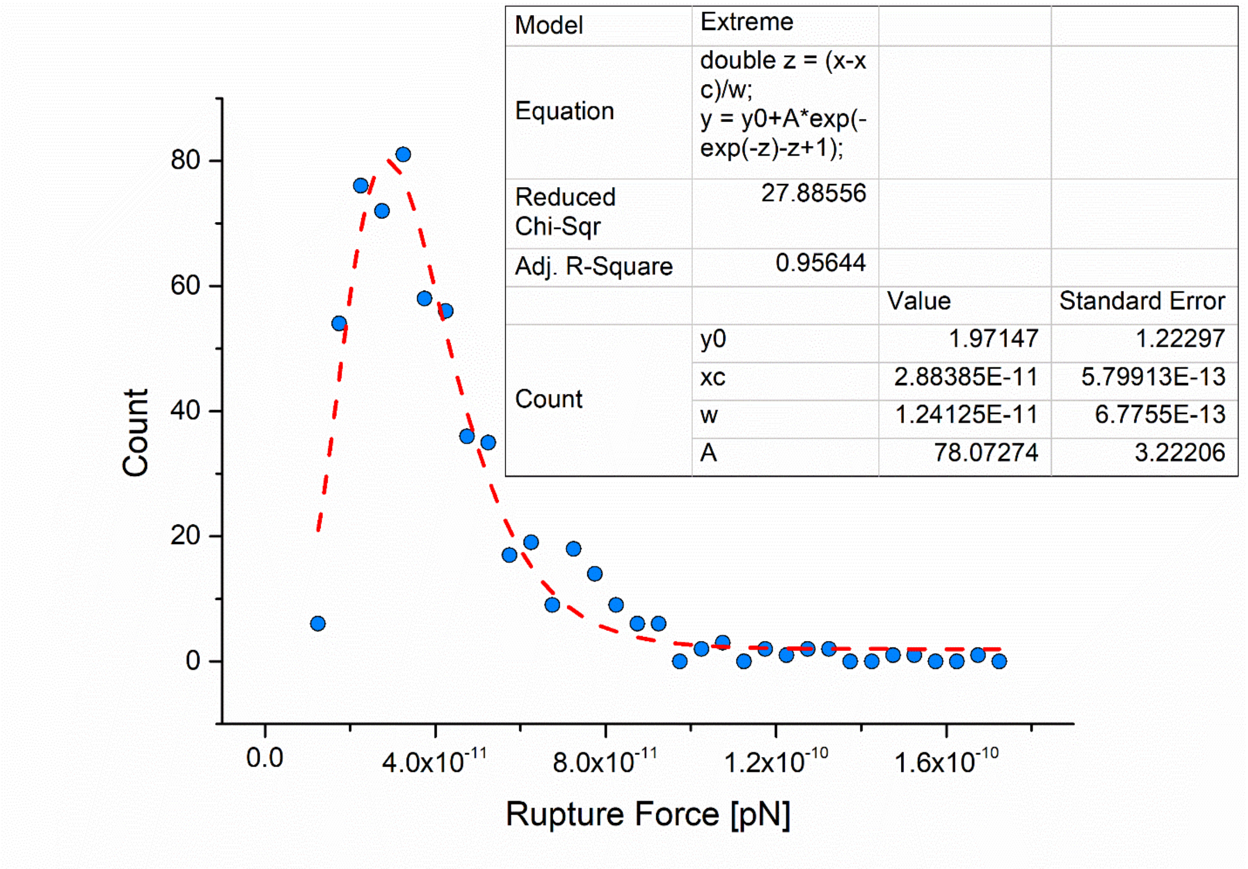

Figure S16. Distribution of rupture forces (after previous counting) and fitting of the distribution with an extreme value function.

S17. Number of rupture events, numerical values and statistical analysis

Table S17 - 1. Number of rupture events, mean value with standard error of mean for Control, 0.1 and 2 mM incubation with colchicine.

	Control	0.1 mM	2 mM
**0**	7.97 ± 0.34	8.33 ± 0.37	8.20 ± 0.59
**60**	7.98 ± 0.43	8.12 ± 0.46	6.92 ± 0.75
**120**	7.72 ± 0.36	5.44 ± 0.43	6.16 ± 0.51
**180**	7.52 ± 0.49	4.53 ± 0.34	5.09 ± 0.40
**240**	8.44 ± 0.36	4.62 ± 0.30	4.45 ± 0.39

Table S17 – 2. Statistical analysis of value evolution over time.

Control	Control	0.1 mM	0.1 mM	2 mM	2 mM
**0**		0		0	
**60**	n.s.	60	n.s.	60	n.s.
**120**	n.s.	120	***	120	n.s.
**180**	n.s.	180	n.s.	180	n.s.
**240**	n.s.	240	n.s.	240	n.s.

Table S17 - 3. Statistical analysis of values over each other.

Control	0.1 mM	Control	2 mM	0.1 mM	2 mM
**0**	n.s.	**0**	n.s.	**0**	n.s.
**60**	n.s.	**60**	n.s.	**60**	*
**120**	***	**120**	*	**120**	n.s.
**180**	***	**180**	***	**180**	n.s.
**240**	***	**240**	***	**240**	n.s.

S18. Rupture position and rupture force

Table S18 - 1. Rupture position (in µm), value derived from extreme value fitting with associated error for Control, 0.1 and 2 mM incubation with colchicine.

	Control	0.1 mM	2 mM
**0**	2.99 ±0.03	3.1 ± 0.03	2.98 ± 0.09
**60**	2.97 ± 0.03	2.05 ± 0.03	2.52 ± 0.04
**120**	2.86 ± 0.03	2.35 ± 0.02	2.58 ± 0.05
**180**	2.85 ± 0.03	2.09 ± 0.05	2.16 ± 0.03
**240**	2.84 ± 0.04	1.66 ± 0.05	1.91 ± 0.02

Table S18 - 2. Rupture force (in pN), value derived from extreme value fitting with associated error for Control, 0.1 and 2 mM incubation with colchicine.

	Control	0.1 mM	2 mM
**0**	32.4 ± 0.73	34.2 ± 0.81	31.5 ± 0.66
**60**	34.8 ± 0.72	37.3 ± 0.87	32.7 ± 0.69
**120**	34.9 ± 0.88	36.6 ± 0.79	34.1 ± 0.84
**180**	32.98 ± 0.54	35.8 ± 0.85	30.5 ± 0.78
**240**	33.66 ± 0.74	33.6 ± 0.91	37.1 ± 0.88

S19 – Raw Data Young's Modulus

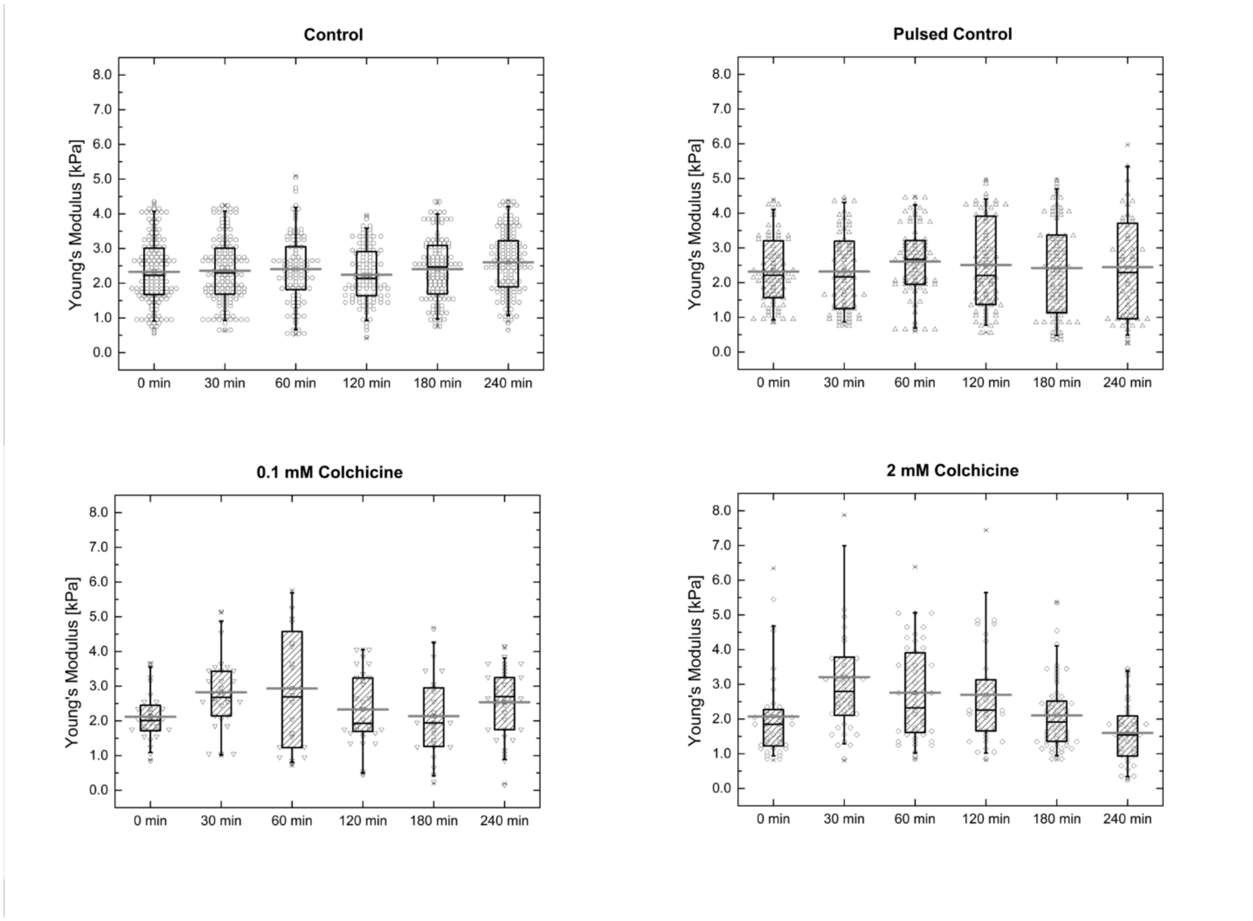

Figure S19. Raw data for Young's Modulus for control, pulsed control, 0.1 mM Colchicine and 2 mM Colchicine. Grey thick line indicates mean value. Box size is 25th to 75th percentile, whisker is 1st to 99th percentile.

S20 – Raw Data Stress relaxation

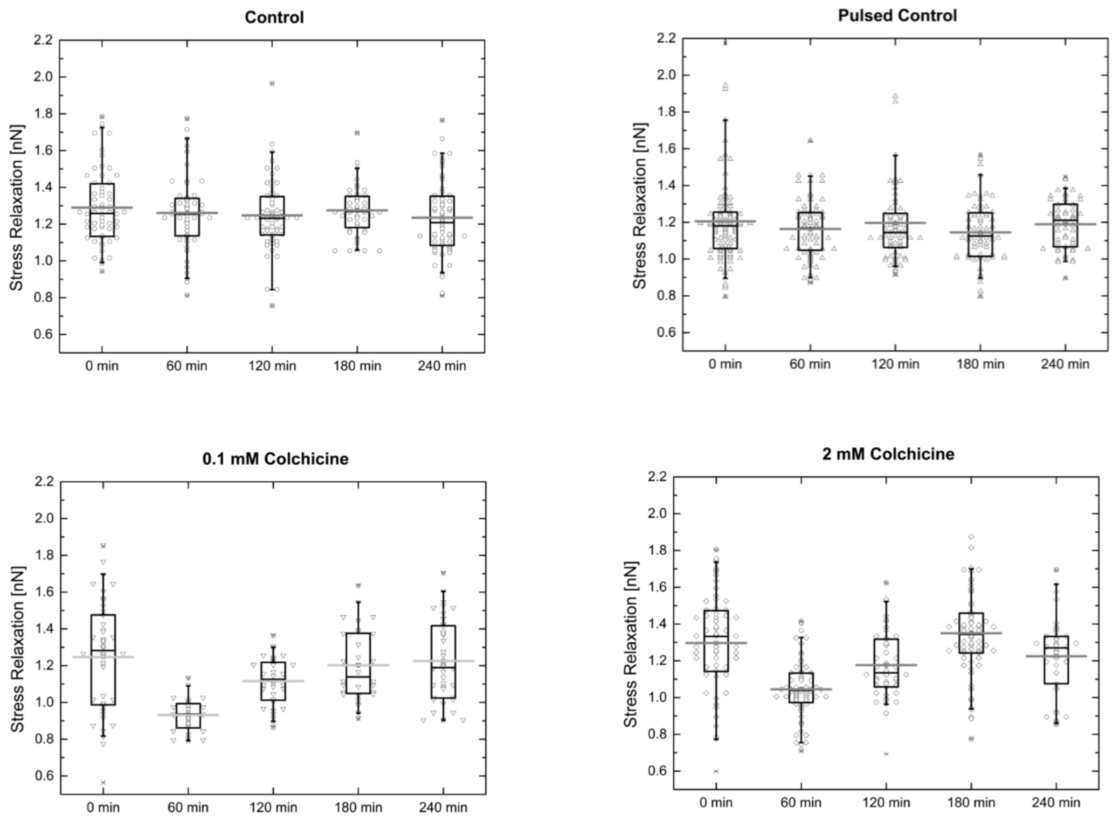

Figure S20. Raw data for stress relaxation for control, pulsed control, 0.1 mM Colchicine and 2 mM Colchicine. Grey thick line indicates mean value. Box size is 25th to 75th percentile, whisker is 1st to 99th percentile.

S21 – Raw data τ_1

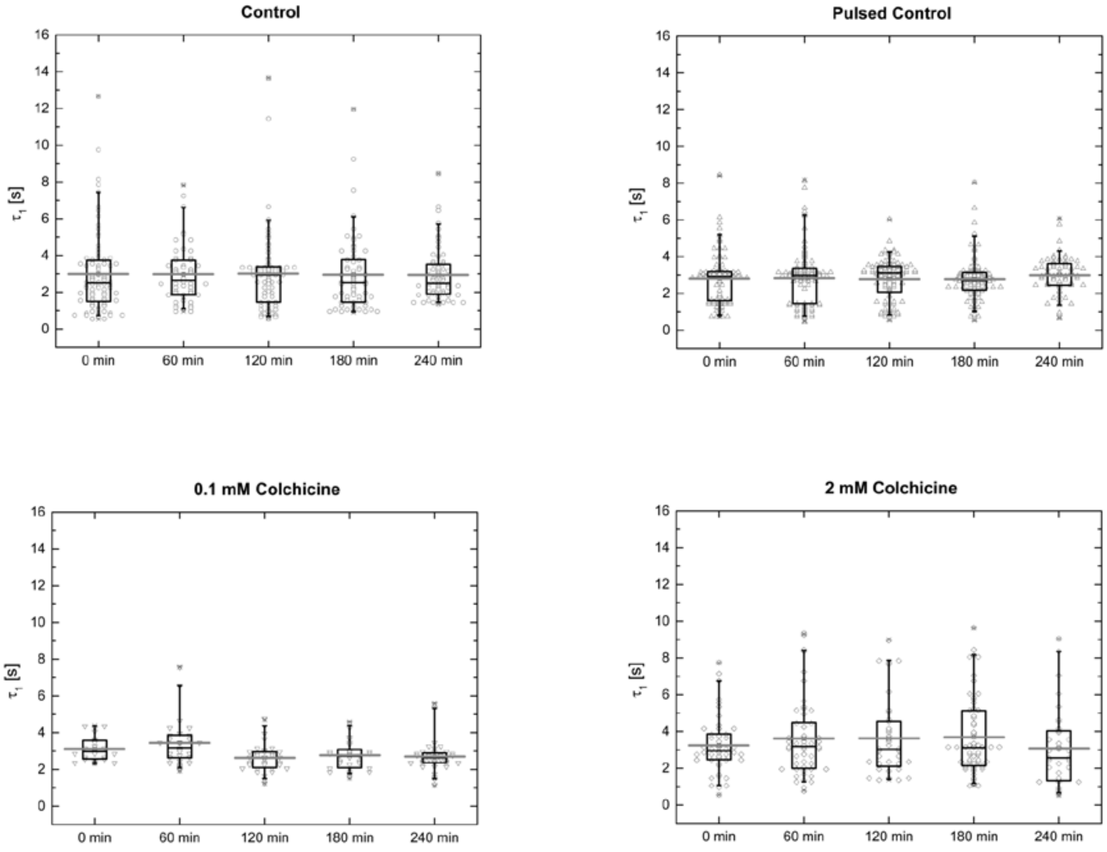

Figure S21. Raw data for τ_1 for control, pulsed control, 0.1 mM Colchicine and 2 mM Colchicine. Grey thick line indicates mean value. Box size is 25th to 75th percentile, whisker is 1st to 99th percentile.

S22 – Raw Data τ_2

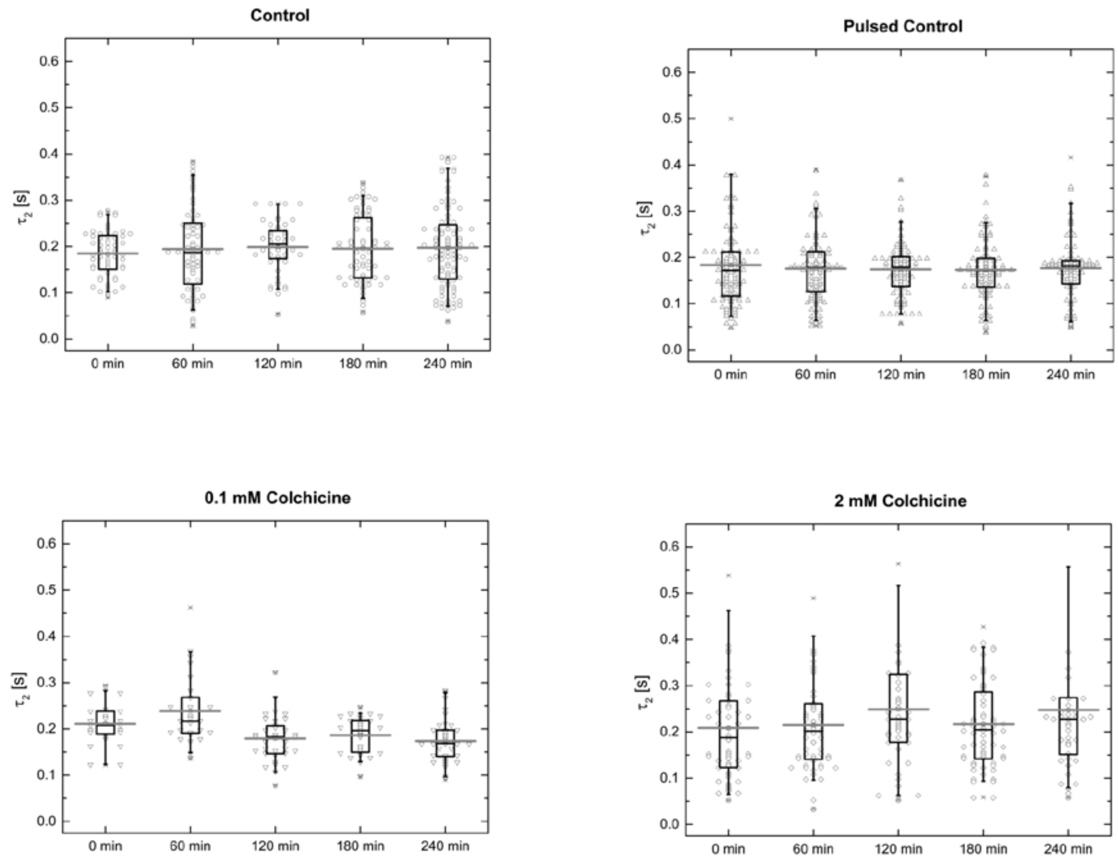

Figure S22. Raw data for τ_2 for control, pulsed control, 0.1 mM Colchicine and 2 mM Colchicine. Grey thick line indicates mean value. Box size is 25th to 75th percentile, whisker is 1st to 99th percentile.

S23 – Raw Data Adhesion between Tip and Cell

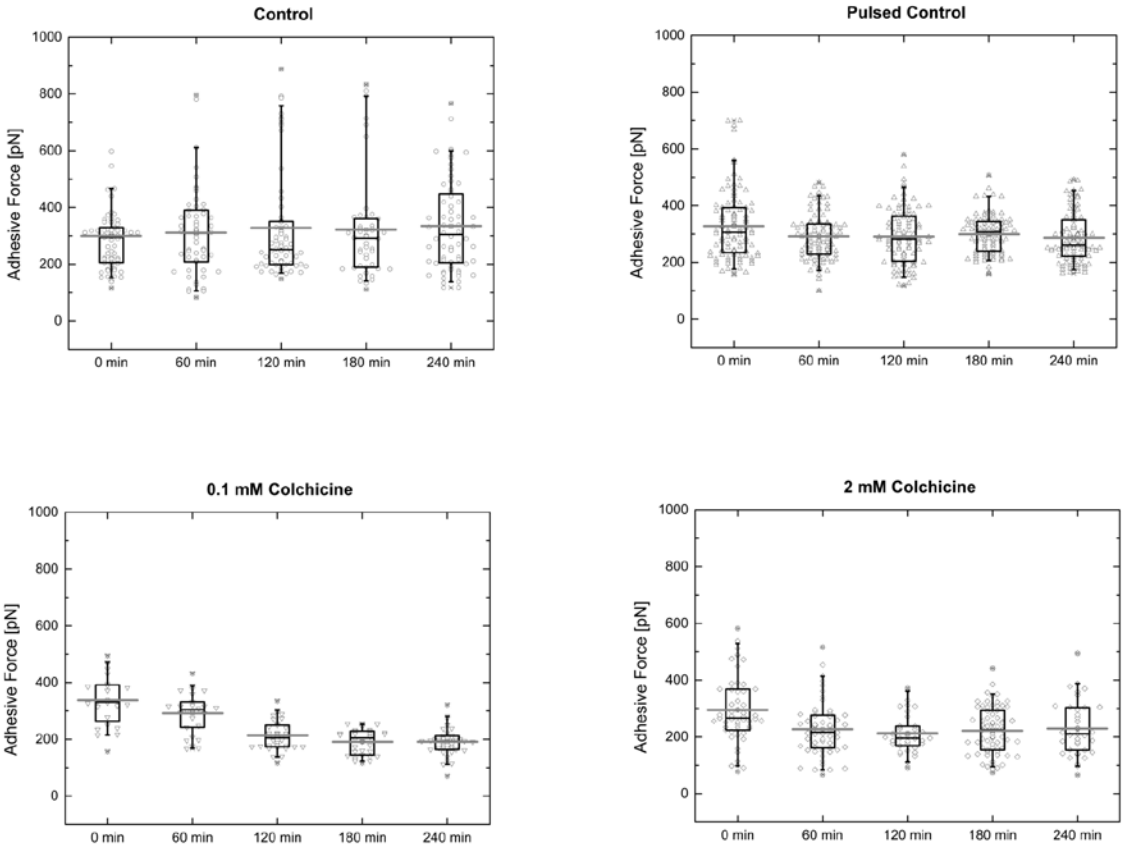

Figure S23. Raw data for adhesion between tip and cell for control, pulsed control, 0.1 mM Colchicine and 2 mM Colchicine. Grey thick line indicates mean value. Box size is 25th to 75th percentile, whisker is 1st to 99th percentile.

S24 – Raw Data number of Events

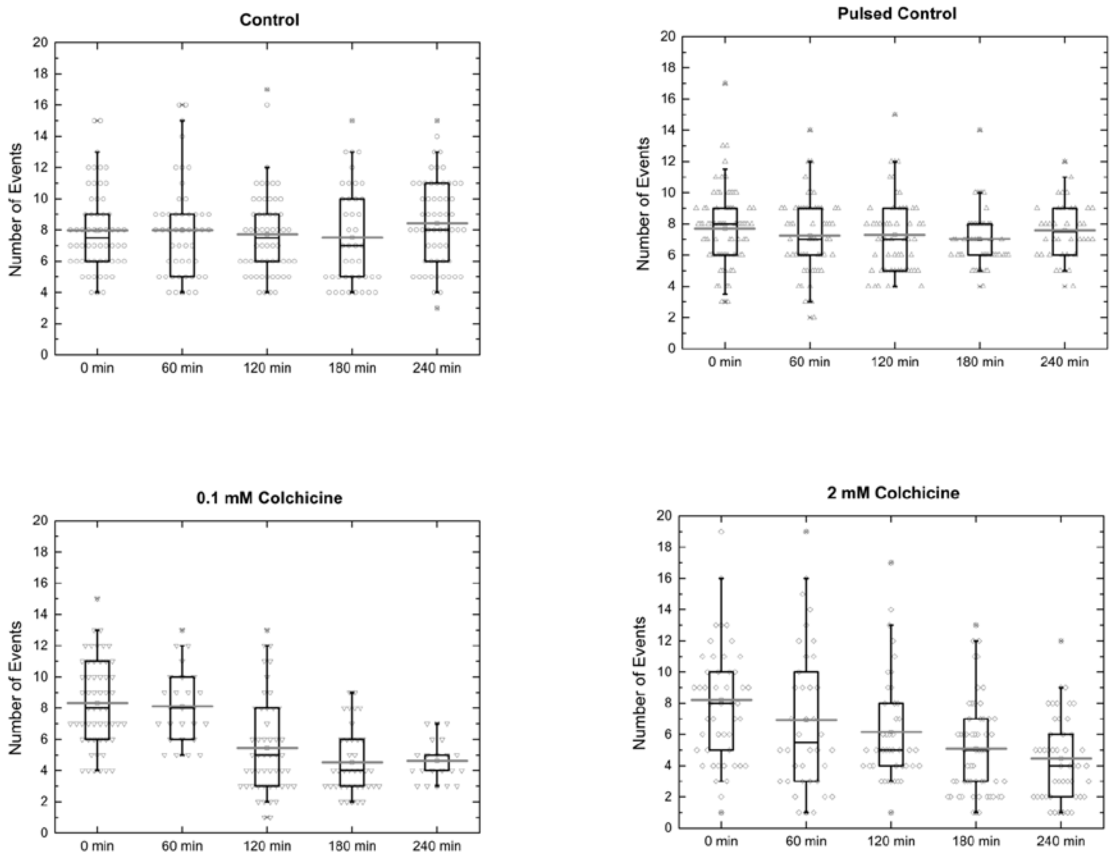

Figure S24. Raw data for number of rupture events for control, pulsed control, 0.1 mM Colchicine and 2 mM Colchicine. Grey thick line indicates mean value. Box size is 25th to 75th percentile, whisker is 1st to 99th percentile.

S25 – Raw Data Position of Rupture Events

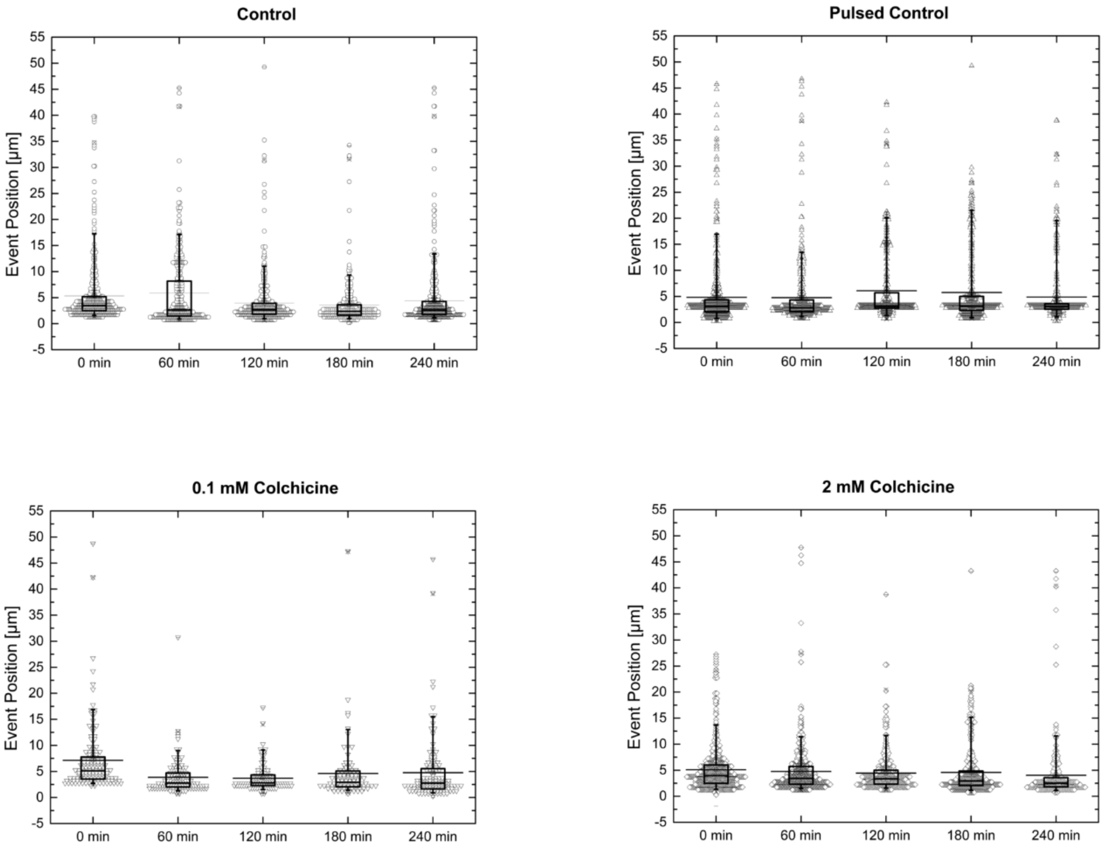

Figure S25. Raw data for rupture event position for control, pulsed control, 0.1 mM Colchicine and 2 mM Colchicine. Grey thick line indicates mean value. Box size is 25th to 75th percentile, whisker is 1st to 99th percentile.

S26 – Raw Data Force of Rupture Events

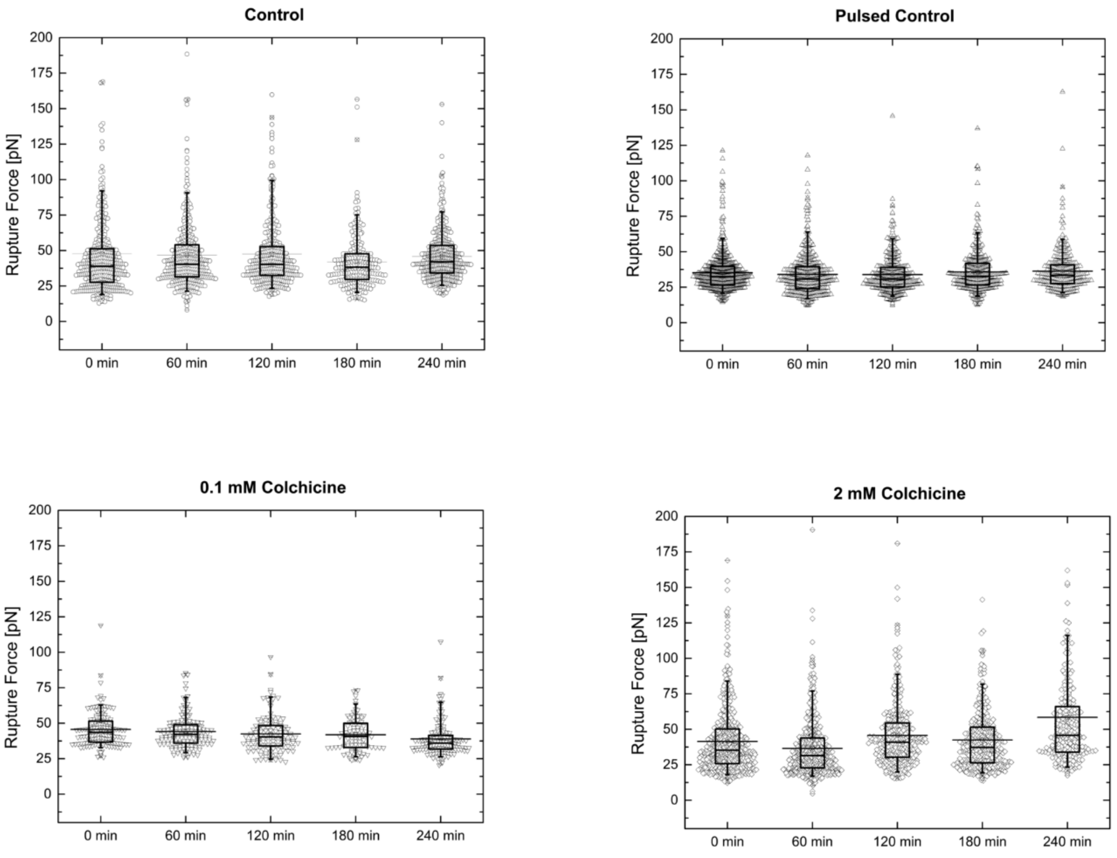

Figure S26. Raw data for rupture force for control, pulsed control, 0.1 mM Colchicine and 2 mM Colchicine. Grey thick line indicates mean value. Box size is 25th to 75th percentile, whisker is 1st to 99th percentile.

S27 – Condensed figures with all data-sets

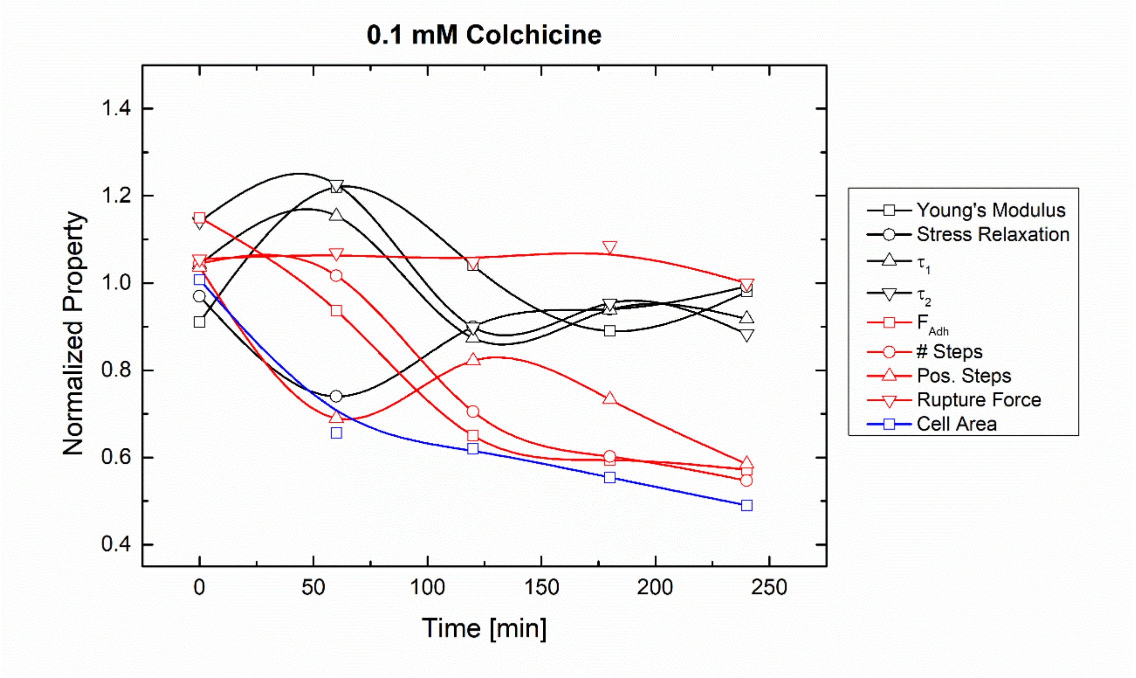

Figure S27.1. Development of the derived parameters for an incubation with 0.1 mM Colchicine. The data shown are normalized by the control values.

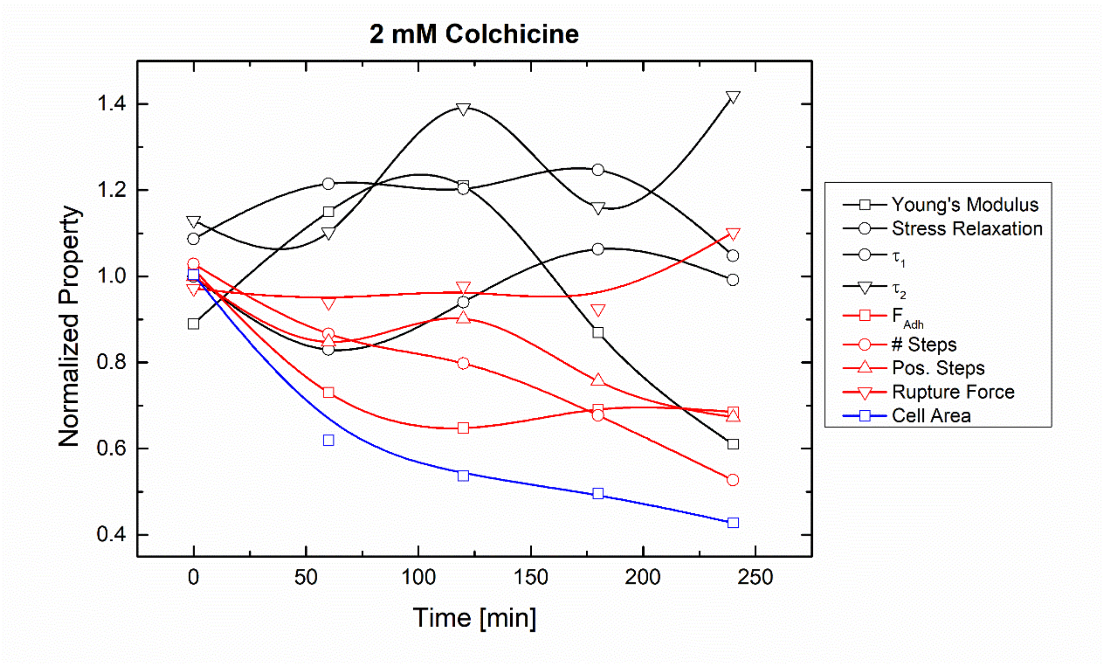

Figure S27.2. Development of the derived parameters for an incubation with 0.1 mM Colchicine. The data shown are normalized by the control values.
